# Synthesis of Phenolic Compounds by Trapping Arynes with a Hydroxy Surrogate

**DOI:** 10.3390/molecules200915862

**Published:** 2015-08-31

**Authors:** Rajdip Karmakar, Sourav Ghorai, Yuanzhi Xia, Daesung Lee

**Affiliations:** 1 Department of Chemistry, University of Illinois at Chicago, 845 West Taylor Street, Chicago, IL 60607, USA; E-Mails: rkarma2@uic.edu (R.K.); sghora2@uic.edu (S.G.); 2 College of Chemistry and Materials Engineering, Wenzhou University, Wenzhou 325035, Zhejiang, China; E-Mail: xyz@wzu.edu.cn

**Keywords:** aryne, bis-functionalization, halophenol, silver trifluoroacetate, regioselectivity

## Abstract

Trapping of arynes with various nucleophiles provides a range of heteroatom-functionalized arene derivatives, but the corresponding reaction with water does not provide phenol derivatives. Silver trifluroacetate (AgO_2_CCF_3_) can nicely solve this problem. It was found that in typical organic solvent, AgO_2_CCF_3_ readily reacts with arynes to generate trifluoroacetoxy organosilver arene intermediate, which, upon treating with silica gel, provides phenolic products. This protocol can be extended to the synthesis of α-halofunctionalized phenol derivatives by simply adding NBS (*N*-bromosuccinimides) or NIS (*N*-iodosuccinimides) to the reaction along with silver trifluroacetate, which provided α-bromo or α-iodophenol derivatives in good yield. However, the similar reactions with NCS (*N*-chlorosuccinimides) afforded only the protonated product instead of the expected α-chlorophenols derivatives. Interestingly, substrates containing silyl substituents on 1,3-diynes resulted in α-halotrifluoroacetates rather than their hydrolyzed product. Additionally, trapping the same arynes with other oxygen-based nucleophiles containing silver counter cation, along with NXS (*N*-halosuccinimides), generated α-halooxyfunctionalized products.

## 1. Introduction

A variety of trapping reactions of arynes [1,2,3,4,5,6,7,8] have been reported on the basis of their highly electrophilic nature [9]. In contrast, a brief screening of literature readily identifies the lack of the examples of aryne trapping with water under traditional aryne formation conditions [10,11,12,13,14,15,16] or under the conditions of the hexadehydro Diels-Alder reaction [17,18,19,20]. Although, in theory, water should be a suitable nucleophile to react with arynes similar to alcohols and carboxylic acids [21,22], the lack of successful trapping of arynes with water might be the consequence of the immiscibility of water with the transient arynes generated in organic solvent, typically CH_2_Cl_2_ or toluene.

**Scheme 1:**
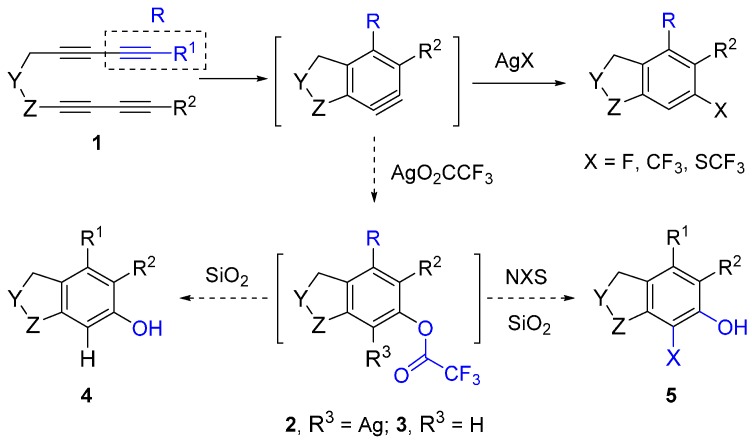
Trapping reactions of an *in situ* generated aryne intermediate with various nucleophiles with a silver counter cation.

It would be highly desirable if we could expand the aryne trapping reaction to directly install a phenolic hydroxyl group on arene scaffolds, as this is an important functionality in large number of compounds, including natural products and pharmaceuticals [23,24]. In search of suitable reagents that can behave like a water surrogate under the given reaction conditions, we refer to a clue suggested by our previous nucleophile trapping study [21,25] of arynes, formed from various tetraynes (**1**), where nucleophiles (F^−^, F_3_C^−^, CF_3_S^−^) associated with a silver counter cation, including silver trifluroacetate (AgO_2_CCF_3_), and provided excellent yields of the corresponding adducts (Scheme 1). Surprisingly, for the similar reaction with silver trifluoroacetate, the protonation of the initially formed putative intermediate **2** did not lead to the expected trifluoroacetate **3**, instead, its deacetylated phenolic product **4** was obtained after purification [21].

## 2. Results and Discussion

On the basis of this initial observation, we carried out a systematic study of aryne trapping reactions with AgO_2_CCF_3_ as a water surrogate to prepare a variety of highly functionalized arene products containing a free phenolic hydroxyl group, and, herein, we report the results.

First, reactions with both symmetrical and unsymmetrical tetrayne substrates of varying substituents were screened to optimize conditions that produce formal water addition products (Table 1). It was quickly identified that the reaction with 1.5 equivalents of AgO_2_CCF_3_ in toluene at 90 °C, followed by purification on silica gel, afforded the phenolic products in good yields. Oxygen-tethered symmetrical tetrayne **1a** with butyl substituents provided a mixture of ortho- and meta-OH adducts ***o*-4a** and ***m*-4a** in a 1.3:1 ratio (Entry 1). The reaction of all-carbon tethered substrate **1b** with a *gem*-dicarboxylate moiety in place of the oxygen tether afforded a similar result, but with slightly improved selectivity and yield (87%) of ***o*-4b** and ***m*-4b** (Entry 2) [26]. Replacing the butyl groups with trimethylsilyl groups afforded only a single isomer ***o-*4c** (Entry 3) [21,27,28,29,30,31,32,33,34,35,36,37]. Although the tether was also changed from oxygen in **1a** to tosylated nitrogen in **1c**, we believe this change has negligible impact on the selectivity. As expected, an ynamide-tethered unsymmetrical tetrayne with triethylsilyl substituents **1d** afforded only the ortho isomer ***o*-4d** in 66% yield (Entry 4). A complete switch in regioselectivity was observed when a tosylated nitrogen tethered symmetrical *bis*-1,3-diyne with phenyl substituents was used, which provided in a majority ***m*-4e** along with ***o*-4e** in a 6.6:1 ratio (entry 5). This switch in regioselectivity can be explained in terms of the charge-controlled model [30], where the electron withdrawing phenyl group creates a more positive character on the farther carbon of the aryne. This allows the nucleophile to attack the meta carbon more preferably. This clearly indicates that, not the tether, but the substituents at the terminal carbon of the 1,3-diyne moieties are the main controlling elements for the selectivity [38].

**Table 1:** Trapping reactions of various aryne intermediate to form phenolic products. 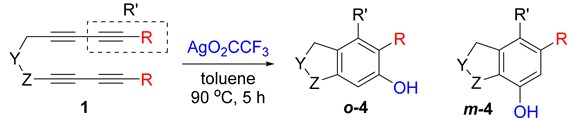

Entry	Diyne	R	Products Ratio ^a^	Yield (%) ^b^
1	**1a**	Bu	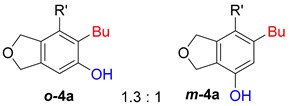	75
2	**1b**	Bu	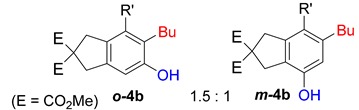	87
3	**1c**	${\mathrm{SiMe}}_{3}$	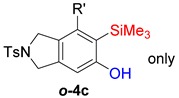	66
4	**1d**	${\mathrm{SiEt}}_{3}$	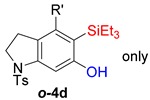	66
5	**1e**	Ph	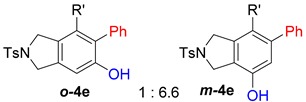	69

^a^ The ratio was determined with the isolated product. ^b^ Isolated yield after SiO_2_ chromatography.

With this result in hand, we envisioned that the putative organosilver intermediate **2** might be captured by suitable electrophiles to generate α-functionalized phenol derivatives. To test the viability of this hypothesis, the reaction was run with *N*-halosuccinimides under otherwise identical conditions, and the results are summarized in Table 2.

**Table 2:** 1,2-Bis functionalization to form α-halophenol derivatives. 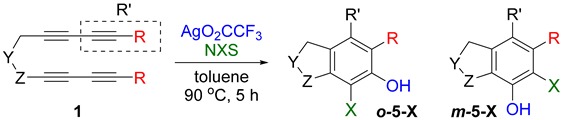

Entry	Diyne	R	Products Ratio ^a^	Yield (%) ^b^
1	**1a**	Bu	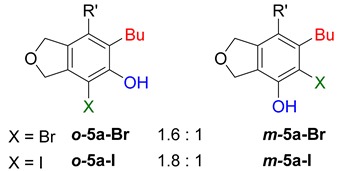	69
2				67
3	**1b**	Bu	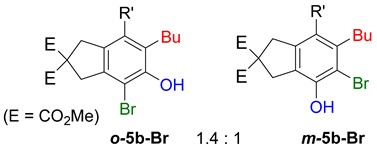	63
4	**1f**	Hex	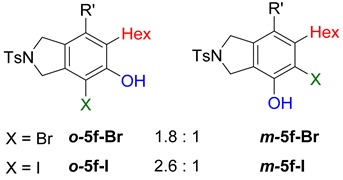	83
5				88
6	**1e**	Ph	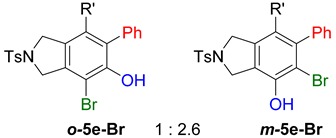	36

^a^ The ratio was determined with the isolated product. ^b^ Isolated yield after SiO_2_ chromatography.

When substrate **1a** was treated with AgO_2_CCF_3_ (1.5 equiv.) and NBS (2.0 equiv.), a mixture of α-bromophenol derivatives ***o-*5a-Br** and ***m-*5a-Br** were obtained in 69% yield with a 1.6:1 ratio (Entry 1). Similarly, with NIS instead of NBS, the corresponding α-iodophenol derivatives ***o-*5a-I** and ***m-*5a-I** were isolated in 67% yield with a 1.8:1 ratio (Entry 2). Substrate **1b** furnished the bromophenol derivatives in 63% yield with an expected selectivity of 1.4:1 [26]. *N*-Tosylamide tethered substrate **1f** containing *n*-hexyl substituents provided bromo and iodophenol derivatives ***o-*5f-Br**/***m-*5f-Br** and ***o-*5a-I/*m-*5a-I** in 83% and 88% yield with a 1.8 and 2.6 ratio, respectively (Entries 5 and 6). Tetrayne **1e** containing phenyl substituents was found to be less efficient and provided a mixture of ***o-*5e-Br** and ***m-*5e-Br** in only 36% yield (Entry 4).

While exploring the scope of the direct synthesis of α-halophenol derivatives, we found that the silyl substituent ortho to the trifluoroacetate moiety interferes with its hydrolysis when halogen was incorporated. Thus, the reaction of **1g** afforded single regioisomer ***o*-6g-CF_3_** as a major product along with expected phenolic product ***o*-5g-Br** in 10% yield (Entry 1) (Table 3). This is in stark contrast to the formation of ***o*-4c** and ***o*-4d**, which are derived from their precursors via complete hydrolysis of the corresponding trifluoroacetates. Based on this observation, we further explored the 1,2-oxyhalogenation to form oxygen-masked form of halophenol derivatives (Table 3). The reaction of substrate **1c** in the presence of silver acetate and NBS provided single regioisomer ***o*-6c-CH_3_** along with phenolic product ***o*-5c-Br** in a 1:2.2 ratio (Entry 2). Unexpectedly, however, the reaction of **1c** with AgO_2_CCF_3_ and NIS afforded a mixture of iodotrifluoroacetates ***o*-6c** and ***m*-6c** in a 6.2:1 ratio devoid of hydrolyzed product (Entry 3). Substrates **1a** and **1f** upon treating with silver triflate and NBS afforded a mixture of bromotriflates ***o*-6a**/***m*-6a** (2.6:1) and ***o*-6f**/***m*-6f** (4.8:1) in 82% and 94% yield, respectively (Entries 4 and 5) [39]. The reaction of **1f** with silver benzoate and NBS provided a mixture of α-bromobenzoates ***o*-6f-Br** and ***m*-6f-Br** in 30% yield with a 2:1 ratio (Entry 6), but, with NCS, not even traces of the expected chloride-trapped product were obtained, instead only protonated products ***o*-6f-H** and ***m*-6f-H** were isolated in 76% yield with a 2.1:1 ratio (Entry 7).

**Table 3:** 1,2-Bis functionalization to form oxygen-masked α-halophenol derivatives. 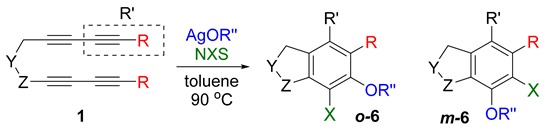

Entry	Diyne	R	Products Ratio ^a^	Yield (%) ^b^
1	**1g**	${\mathrm{SiEt}}_{3}$	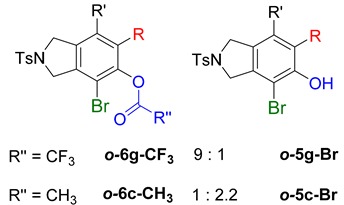	82
2	**1c**	${\mathrm{SiMe}}_{3}$		52
3	**1c**	${\mathrm{SiMe}}_{3}$	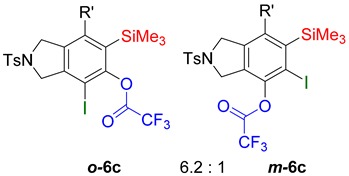	65
4	1a	Bu	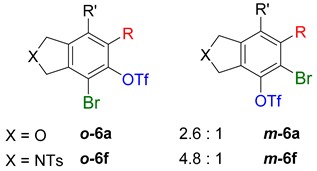	82
5	1f	Hex		94
6	1f	Hex	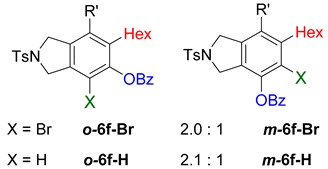	30
7				76

^a^ The ratio was determined with the isolated product. ^b^ Isolated yield after SiO_2_ chromatography.

## 3. Experimental Section

### 3.1. General Information

Reactions were carried out in oven-dried glassware unless otherwise noted. Compounds were purchased from Aldrich, Acros, TCI America, or Oakwood Chemicals, unless otherwise noted. Toluene, acetonitrile, and dichloromethane were distilled over calcium hydride (CaH_2_) under a nitrogen atmosphere. THF was distilled over sodium-benzophenone ketyl under a nitrogen atmosphere. Column chromatography was performed using silica gel 60 Å (32−63 mesh), purchased from Silicycle Inc. (Quebec, QC, Canada). Analytical thin layer chromatography (TLC) was performed on 0.25 mm E. Merck precoated silica gel 60 (particle size 0.040−0.063 mm). Yields refer to chromatographically and spectroscopically pure compounds unless otherwise stated. ^1^H-NMR and ^13^C-NMR spectra were recorded on a Bruker AV-500 spectrometer (Bruker BioSpin Corporation, Billerica, MA, USA). ^19^F-NMR spectrum was recorded in Varian Mercury-Vx-300 spectrometer (Palo Alto, CA, USA). ^1^H-NMR chemical shifts (δ) are reported in parts per million (ppm) downfield of TMS and are referenced relative to the residual proteated solvent peak (CDCl_3_ (7.26 ppm)). ^13^C chemical shifts (δ) are reported in parts per million downfield of TMS and are referenced to the carbon resonance of the solvent (CDCl_3_ (77.2 ppm)). Multiplicities are indicated by s (singlet), d (doublet), t (triplet), q (quartet), quin (quintet), sext (sextet), or m (multiplet). ^1^H-NMR signals that fall within a *ca.* 0.3-ppm range are generally reported as a multiplet, with a range of chemical shift values corresponding to the peak or center of the peak. Coupling constants, *J*, are reported in Hz (Hertz). Electrospray ionization (ESI) mass spectra were recorded on a Waters Micromass Q-Tof Ultima (Waters Corporation, Milford, MA, USA) at the University of Illinois at Urbana-Champaign. Electron impact (EI) mass spectra and Chemical Ionization (CI) mass spectra were obtained using a Micromass 70-VSE (Waters Corporation, Milford, MA, USA) at the University of Illinois at Urbana-Champaign.

### 3.2. Experimental Details

#### 3.2.1. General Procedure for the Mono-Functionalization (GPM)

In a glove box, a mixture of a substrate (0.1 mmol, 1.0 equiv.) and a nucleophile (0.15 mmol, 1.5 equiv.) in dry toluene (3 mL) was taken into a Schlenk tube. The reaction mixture was stirred at 90 °C for 5 h, unless otherwise noted. After completion, the reaction mixture was transferred to a round-bottom flask, concentrated and loaded on silica gel column for chromatographic purification, using ethyl acetate-hexane mixture as the eluent.

#### 3.2.2. General Procedure for the Bis-Functionalization (GPB)

In a glove box, a mixture of a substrate (0.1 mmol, 1.0 equiv.) and a nucleophile (0.15 mmol, 1.5 equiv.) and an electrophile (0.2 mmol, 2.0 equiv.) in dry toluene (3 mL) was taken into a Schlenk tube. The reaction mixture was stirred at 90 °C for 5 h, unless otherwise noted. After completion, the reaction mixture was transferred to a round-bottom flask, concentrated and subjected to column chromatography, using ethyl acetate-hexane mixture as the eluent, to get pure products.

#### 3.2.3. Characterization Data of the Products


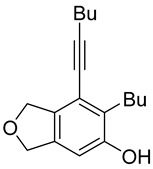

***o*-4a**: This compound was prepared using GPM as an inseparable mixture of isomers (*o*/*m* = 1.3:1) in 75% overall yield after column chromatographic purification. ^1^H-NMR (CDCl_3_, 500 MHz): δ (major isomer) 6.58 (s, 1H), 5.07–5.04 (m, 4H), 4.76 (s, 1H), 2.78 (t, 2H, *J* = 8.0 Hz), 2.45 (t, 2H, *J* = 7.0 Hz), 1.64–1.36 (m, 8H), 0.97–0.92 (m, 6H); ^13^C-NMR (CDCl_3_, 125 MHz): δ (major isomer) 153.1, 137.1, 134.0, 129.6, 118.2, 107.4, 97.5, 76.6, 74.1, 74.0, 31.7, 30.9, 27.8, 22.9, 21.9, 19.2, 14.0, 13.6; HRMS (ESI) calcd for C_18_H_25_O_2_ [M + H]^+^ 273.1849, found 273.1850.

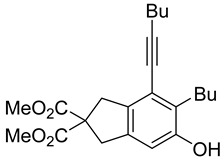

***o*-4b**: This compound was formed through GPM at 120 °C (overnight) in an inseparable mixture of two isomers with 66% overall yield after purification by column chromatography. ^1^H-NMR (CDCl_3_, 500 MHz): δ (major isomer) 6.55 (s, 1H), 4.71 (s, 1H), 3.75 (s, 6H), 3.57 (s, 2H), 3.51 (s, 2H), 2.73 (t, 2H, *J* = 7.8 Hz), 2.46 (t, 2H, *J* = 7.0 Hz), 1.62–1.32 (m, 8H), 0.99–0.89 (m, 6H); ^13^C-NMR (CDCl_3_, 125 MHz): δ (all discernible signals for both isomers) 172.3, 172.1, 152.8, 150.7, 145.9, 144.8, 137.7, 134.6, 129.2, 122.7, 120.8, 114.2, 112.2, 110.6, 110.0, 97.4, 95.8, 59.8, 59.6, 53.0, 53.0, 41.3, 40.8, 40.4, 37.3, 34.0, 32.9, 31.7, 31.1, 31.0, 28.0, 22.9, 22.6, 22.0, 19.3, 14.0, 13.95, 13.6; HRMS (ESI) calcd for C_23_H_31_O_5_ [M + H]^+^ 387.2166, found 387.2162.

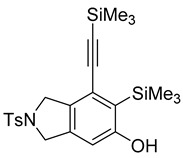

***o*-4c**: This compound was prepared using GPM. only single isomer was isolated in 66% after purification using column chromatography. ^1^H-NMR (CDCl_3_, 500 MHz) δ 7.77–7.74 (m, 2H), 7.33–7.30 (m, 2H), 6.49 (s, 1H), 5.28 (s, 1H), 4.55 (s, 4H), 2.41 (s, 3H), 0.41 ( s, 9H), 0.25 (s, 9H); ^13^C-NMR (CDCl_3_, 125 MHz): δ 160.57, 143.7, 138.3, 133.9, 132.8, 129.8, 127.6, 125.4, 124.8, 109.8, 103.4, 103.0, 54.3, 53.8, 21.5, 1.1, −0.3; HRMS (ESI) calcd for C_23_H_32_NO_3_SSi_2_ [M + H]^+^ 458.1636, found 458.1624.

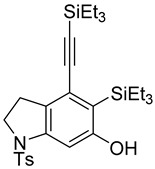

***o*-4d**: This compound was prepared using GPM and isolated in 66% yield as a single isomer after column chromatographic purification. ^1^H-NMR (CDCl_3_, 500 MHz): δ 7.73–7.69 (m, 2H), 7.28–7.24 (m, 2H), 7.02 (s, 1H), 5.09 (s, 1H), 3.88 (t, 2H, *J* = 10 Hz), 2.92 (t, 2H, *J* = 10 Hz), 2.39 (s, 3H), 1.03–0.91 (m, 24H), 0.67–0.57 (m, 6H); ^13^C-NMR (CDCl_3_, 125 MHz): δ 161.6, 144.3, 143.5, 134.0, 129.8, 128.6, 127.3, 126.7, 117.2, 104.8, 102.3, 99.9, 49.9, 27.9, 21.6, 7.7, 7.4, 4.7, 4.3; HRMS (ESI) calcd for C_29_H_44_NO_3_SSi_2_ [M + H]^+^ 542.2575, found 542.2571.

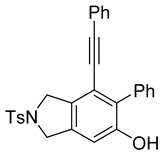

***o*-4e**: This compound was produced through GPM with other isomer with 69% overall yield after purification by column chromatography. ^1^H-NMR (CDCl_3_, 500 MHz): δ (major isomer) 7.83–7.80 (m, 2H), 7.59–7.55 (m, 2H) 7.43–7.28 (m, 10H), 6.74 (s, 1H), 5.74 (s, 1H), 4.79 (m, 2H), 4.69 (m, 2H), 2.41 (s, 3H); ^13^C-NMR (CDCl_3_, 125 MHz): δ (major isomer) 150.6, 145.6, 143.8, 141.8, 139.5, 133.8, 131.3, 130.0, 129.1, 128.3, 128.2 128.0, 127.8, 127.6, 123.2, 121.8, 115.8, 109.0, 95.0, 85.8, 54.7, 52.3, 21.5; HRMS (ESI) calcd for C_29_H_24_NO_3_S [M + H]^+^ 466.1471, found 466.1469.

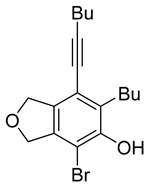

***o*-5a-Br**: This compound was prepared using GPB and isolated in 42% yield after purification by column chromatography. ^1^H-NMR (CDCl_3_, 500 MHz): δ 5.45 (s, 1H), 5.17–5.15 (m, 2H), 5.07–5.04 (m, 2H), 2.86–2.81 (m, 2H), 2.44 (t, 2H, *J* = 6.8 Hz), 1.62–1.36 (m, 8H), 0.95 (t, 3H, *J* = 7.3 Hz), 0.94 (t, 3H, *J* = 7.3 Hz); ^13^C-NMR (CDCl_3_, 125 MHz): δ 149.4, 136.7, 134.3, 131.6, 117.6, 103.0, 98.2, 76.1, 75.5, 75.4, 31.5, 30.8, 28.8, 22.8, 21.9, 19.3, 14.0, 13.6.

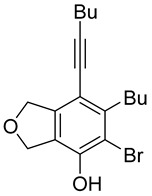

***m*-5a-Br**: This compound was prepared using GPB and isolated in 27% yield after purification by column chromatography. ^1^H-NMR (CDCl_3_, 500 MHz): δ 5.77 (s, 1H), 5.14 (m, 2H), 5.09 (m, 2H), 2.92 (t, 2H, *J* = 8.1 Hz), 2.44 (t, 2H, *J* = 6.9 Hz), 1.61–1.40 (m, 8H), 0.99–0.92 (m, 6H); ^13^C-NMR (CDCl_3_, 125 MHz): δ 145.9, 144.1, 143.5, 122.9, 111.2, 110.3, 96.3, 76.1, 74.5, 72.8, 34.8, 31.3, 30.9, 22.8, 21.9, 19.2, 13.9, 13.6; HRMS (ESI) calcd for C_18_H_22_BrO_2_ [M − H]^+^ 349.0803, found 349.0800.

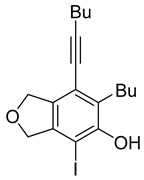

***o*-5a-I**: This compound was prepared using GPB and isolated in 43% yield after separation using column chromatography. ^1^H-NMR (CDCl_3_, 500 MHz): δ 5.23–5.20 (m, 3H), 4.99–4.96 (m, 2H), 2.85 (t, 2H, *J* = 7.7 Hz), 2.44 (t, 2H, *J* = 6.9 Hz), 1.62–1.36 (m, 8H), 0.98–0.92 (m, 6H); ^13^C-NMR (CDCl_3_, 125 MHz): δ 151.8, 140.8, 134.1, 130.4, 118.7, 98.5, 78.6, 78.3, 76.0, 75.9, 31.5, 30.8, 29.2, 22.8, 21.9, 19.3, 14.0, 13.6; HRMS (ESI) calcd for C_18_H_22_IO_2_ [M − H]^+^ 397.0664, found 397.0660.

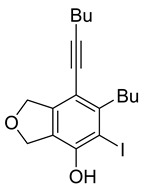

***m*-5a-I**: This compound was prepared using method B and isolated in 24% yield after column chromatographic separation. ^1^H-NMR (CDCl_3_, 500 MHz): δ 5.61 (s, 1H), 5.14–5.12 (m, 2H), 5.11–5.09 (m, 2H), 2.99–2.94 (m, 2H), 2.45 (t, 2H, *J* = 6.5 Hz), 1.62–1.41 (m, 8H), 1.01–0.91 (m, 6H); ^13^C-NMR (CDCl_3_, 125 MHz): δ 148.2, 147.4, 144.8, 122.1, 109.8, 96.2, 92.0, 76.3, 74.4, 73.0, 39.6, 31.3, 30.9, 22.9, 21.9, 19.2, 13.9, 13.6; HRMS (ESI) calcd for C_18_H_24_IO_2_ [M + H]^+^ 399.0815, found 399.0803.

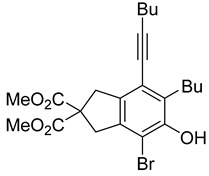

***o*-5b-Br**: This compound was prepared using GPB at 120 °C (overnight) and isolated in 63% yield after column chromatographic purification. ^1^H-NMR (CDCl_3_, 500 MHz): δ 5.38 (s, 1H), 3.77 (s, 6H), 3.68 (s, 2H), 3.58 (s, 2H), 2.80 (t, 2H, *J* = 8.0 Hz), 2.46 (t, 2H, *J* = 7.0 Hz), 1.64–1.34 (m, 8H), 0.96 (t, 3H, *J* = 7.5 Hz), 0.93 (t, 3H, *J* = 8.0 Hz); ^13^C-NMR (CDCl_3_, 125 MHz): δ 171.9, 149.1, 137.1, 134.7, 131.1, 120.2, 106.6, 98.2, 76.7, 53.1, 42.6, 41.5, 31.5, 30.9, 29.0, 22.8, 22.0, 19.4, 14.0, 13.6; HRMS (ESI) calcd for C_23_H_30_BrO_5_ [M + H]^+^ 465.1271, found 465.1184.

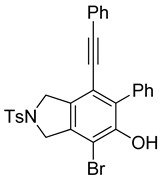

***o*-5e-Br**: This compound was prepared using GPB and isolated in inseparable mix with 36% overall yield after isolation using column chromatography. ^1^H-NMR (CDCl_3_, 500 MHz): δ 7.85–7.82 (m, 2H), 7.48–7.42 (m, 3H), 7.37–7.33 (m, 2H), 7.31–7.27 (m, 3H), 7.26–7.22 (m, 2H), 7.11–7.08 (m, 2H), 5.94 (s, 1H), 4.79–4.76 (m, 2H), 4.74–4.71 (m, 2H), 2.43 (s, 3H); ^13^C-NMR (CDCl_3_, 125 MHz): δ (all discernible signals for both isomers) 148.0, 147.9, 147.6, 147.3, 145.3, 143.9, 143.9, 139.9, 139.3, 138.1, 136.4, 133.8, 131.3, 130.0, 129.6, 129.4, 129.4, 129.2, 129.0, 128.9, 128.8, 128.6 , 128.4, 128.3, 128.3, 128.2, 128.0, 127.9, 127.8, 127.6, 122.8, 122.6, 122.3, 122.1, 111.7, 111.0, 96.0, 84.9, 54.5, 53.4, 52.5, 52.3, 21.5; HRMS (ESI) calcd for C_29_H_23_BrNO_3_S [M + H]^+^ 544.0577, found 544.0576.

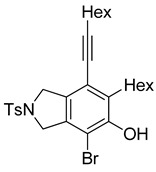

***o*-5f-Br**: This compound was prepared using GPB and isolated in 61% yield after purification by column chromatography. ^1^H-NMR (CDCl_3_, 500 MHz): δ 7.79–7.76 (m, 2H), 734–7.31 (m, 2H), 5.41 (s, 1H), 4.67–4.64 (m, 2H), 4.56–4.54 (m, 2H), 2.79–2.74 (m, 2H), 2.44 (t, 2H, *J* = 7.0 Hz), 2.41 (s, 3H), 1.64–1.57 (m, 2H), 1.54–1.42 (m, 4H), 1.38–1.25 (m, 10 H), 0.92 (m, 3H), 0.90–0.85 (m, 3H); ^13^C-NMR (CDCl_3_, 125 MHz): δ 149.7, 143.7, 133.9, 133.7, 132.1, 131.2, 129.9, 127.6, 119.1, 104.2, 99.3, 75.6, 55.7, 55.0, 31.7, 31.4, 29.4, 29.12, 29.09, 28.7, 28.6, 22.6, 21.5, 19.6, 14.1; HRMS (ESI) calcd for C_29_H_39_BrNO_3_S [M + H]^+^ 560.1829, found 560.1829.

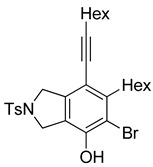

***m*-5f-Br**: This compound was prepared using method GPB and isolated in 22% yield after purification by column chromatography. ^1^H-NMR (CDCl_3_, 500 MHz): δ 7.79–7.76 (m, 2H), 7.33–7.30 (m, 2H), 5.74 (s, 1H), 4.60 (s, 4H), 2.88–2.83 (m, 2H), 2.46–2.42 (m, 2H), 2.41 (s, 3H), 1.63–1.54 (t, 3H), 1.53–1.42 (m, 3H), 1.41–1.27 (m, 10H), 0.94–0.86 (m, 6H); ^13^C-NMR (CDCl_3_, 125 MHz): δ 146.5, 144.6, 143.7, 140.0, 133.9, 129.9, 127.6, 120.2, 111.8, 111.4, 97.3, 75.6, 54.5, 52.4, 35.2, 31.6, 31.4, 29.4, 29.0, 28.8, 28.6, 22.6, 21.5, 19.6, 14.1; HRMS (ESI) calcd for C_29_H_39_BrNO_3_S [M + H]^+^ 560.1829, found 560.1829.

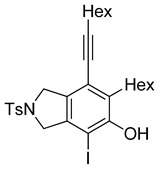

***o*-5f-I**: This compound was prepared using GPB and isolated in 64% yield after purification by column chromatography. ^1^H-NMR (CDCl_3_, 500 MHz): δ 7.80–7.76 (m, 2H), 7.34–7.31 (m, 2H), 5.19 (s, 1H), 4.71–4.69 (m, 2H), 4.50–4.47 (m, 2H), 2.81–2.76 (m, 2H), 2.44 (t, 2H, *J* = 7.0 Hz), 2.41 (s, 3H), 1.64–1.42 (m, 6H), 1.38–1.24 (m, 10H), 0.92 (t, 3H, *J* = 6.8 Hz), 0.89–0.85 (m, 3H); ^13^C-NMR (CDCl_3_, 125 MHz): δ 152.2, 143.7, 137.8, 133.9, 131.0, 130.9, 130.0, 129.9, 127.6, 120.1, 99.6, 80.3, 75.5, 59.1, 55.4, 31.7, 31.4, 29.7, 29.5, 29.4, 29.1, 28.7, 28.6, 22.6, 21.5, 19.6, 14.1; HRMS (ESI) calcd for C_29_H_39_INO_3_S [M + H]^+^ 608.1690, found 608.1694.

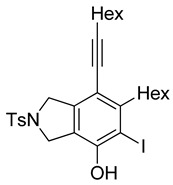

***m*-5f-I**: This compound was isolated in 24% yield using GPB after column chromatographic separation. ^1^H-NMR (CDCl_3_, 500 MHz): δ 7.79–7.76 (m, 2H), 7.33–7.30 (m, 2H), 5.58 (s, 1H), 4.63–4.59 (m, 4H), 2.92–2.87 (m, 2H), 2.44 (t, 2H, *J* = 7.2 Hz), 2.40 (s, 3H), 1.63–1.29 (m, 16H), 0.94–0.87 (m, 6H); ^13^C-NMR (CDCl_3_, 125 MHz): δ 148.9, 147.9, 143.7, 141.3, 133.9, 129.9, 127.6, 119.3, 111.2, 97.2, 91.3, 75.8, 54.5, 52.7, 40.1, 31.5, 31.4, 29.4, 29.0, 28.8, 28.6, 22.6, 21.5, 19.6, 14.1; HRMS (ESI) calcd for C_29_H_39_INO_3_S [M + H]^+^ 608.1690, found 608.1692.

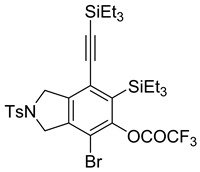

***o*-6g-CF_3_**: This compound was prepared using GPB and isolated in 72% yield after column chromatographic purification. ^1^H-NMR (CDCl_3_, 500 MHz): δ 7.78–7.75 (m, 2H), 7.36–7.32 (m, 2H), 4.79–4.58 (m, 4H), 2.42 (s, 3H), 1.08–0.95 (m, 12H), 0.91–0.86 (m, 12H), 0.71 (q, 6H, *J* = 7.9 Hz); ^13^C-NMR (CDCl_3_, 125 MHz): δ 156.2 (C=O), 155.8 (C=O), 155.5 (C=O), 155.1 (C=O), 151.0, 144.1, 141.3, 140.2, 133.5, 133.1, 130.0, 127.6, 124.5, 115.6 (CF_3_), 113.4(CF_3_), 111.2, 104.8, 102.3, 56.2, 55.4, 21.5, 7.4, 7.3, 4.1, 4.0; HRMS (ESI) calcd for C_31_H_42_BrF_3_NO_4_SSi_2_ [M + H]^+^ 716.1503, found 716.1504.

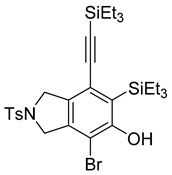

***o*-5g-Br**: This compound was isolated in 10% after column chromatographic purification in GPB. ^1^H-NMR (CDCl_3_, 500 MHz): δ 7.77–7.74 (m, 2H), 7.34–7.31 (m, 2H), 5.64 (s, 1H), 4.64–4.58 (m, 4H), 2.41 (s, 3H), 1.07–0.95 (m, 15H), 0.94–0.89 (m, 9H), 0.69 (q, 6H, *J* = 6.9 Hz). HRMS (ESI) calcd for C_29_H_43_BrNO_3_SSi_2_ [M + H]^+^ 620.1680, found 620.1669.

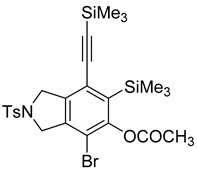

***o*-6d-CH_3_**: This compound was prepared using GPB and isolated in 16% yield after purification by column chromatography. ^1^H-NMR (CDCl_3_, 500 MHz): δ 7.79–7.76 (m, 2H), 7.36–7.33 (m, 2H), 4.8–4.53 (m, 4H), 2.43 (s, 3H), 2.31 (s, 3H), 0.36 (s, 9H), 0.25 (s, 9H); ^13^C-NMR (CDCl_3_, 125 MHz): δ 169.0, 151.8, 144.0, 139.5, 139.2, 135.8, 133.6, 130.0, 127.5, 123.6, 112.7, 105.8, 101.7, 56.1, 55.1, 21.5, 21.3, 0.7, −0.4; HRMS (ESI) calcd for C_25_H_33_BrNO_4_SSi_2_ [M + H]^+^ 578.0847, found 578.0846.

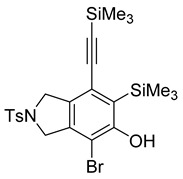

***o*-5d-Br**: This compound was prepared using GPB and isolated in 36% yield after purification by column chromatography. ^1^H-NMR (CDCl_3_, 500 MHz): δ 7.79–7.76 (m, 2H), 7.35–7.32 (m, 2H), 5.66 (s, 1H), 4.66–4.64 (m, 2H), 4.58–4.56 (m, 2H), 2.42 (s, 3H), 0.38 (s, 9H), 0.25 (s, 9H); ^13^C-NMR (CDCl_3_, 125 MHz): δ 155.9, 143.8, 137.9, 133.8, 133.5, 129.9, 127.5, 127.5, 123.5, 105.8, 104.1, 102.2, 56.0, 55.0, 21.5, 1.1, −0.4; HRMS (ESI) calcd for C_23_H_31_BrNO_3_SSi_2_ [M + H]^+^ 536.0741, found 536.0740.

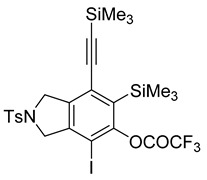

***o*-6d**: This compound was prepared using GPB and isolated in 48% yield after purification by column chromatography. ^1^H-NMR (CDCl_3_, 500 MHz): δ 7.80–7.77 (m, 2H), 7.37–7.34 (m, 2H), 4.86–4.68 (m, 2H), 4.63–4.49 (m, 2H), 2.43 (s, 3H), 0.36 (s, 9H), 0.26 (s, 9H); ^13^C-NMR (CDCl_3_, 125 MHz): δ 156.0 (C=O), 155.7 (C=O), 152.9, 144.7, 144.1, 139.7, 134.7, 133.6, 130.1, 127.5, 125.2, 118.0 (CF_3_), 115.7 (CF_3_), 113.5 (CF_3_), 107.2, 101.2, 86.4, 59.7, 55.4, 21.5, 0.6, −0.5; HRMS (ESI) calcd for C_25_H_30_F_3_INO_4_SSi_2_ [M + H]^+^ 680.0425, found 680.0433.

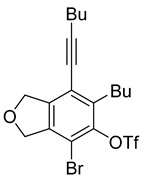

***o*-6a**: This compound was prepared using method GPB in an inseparable mixture of two isomers (2.7:1) with 83% overall yield after column chromatography purification. ^1^H-NMR (CDCl_3_, 500 MHz): δ (major isomer) 5.22–5.20 (m, 2H), 5.11–5.09 (m, 2H), 2.94–2.90 (m, 2H), 2.46 (t, 2H, *J* = 6.5 Hz), 1.64–1.36 (m, 8H), 0.98–0.92 (m, 6H); ^13^C-NMR (CDCl_3_, 500 MHz): δ (all discernible signals for both isomers) 146.7, 144.6, 143.9, 142.8, 139.8, 139.6, 139.4, 130.9, 130.1, 129.9, 129.7, 129.6, 128.2, 128.1, 127.9, 126.0, 125.6, 119.9, 118.8, 118.6, 117.4, 116.8, 108.9, 100.7, 100.4, 75.8, 75.3, 74.5, 72.8, 35.1, 35.1, 31.6, 31.0, 30.6, 30.6, 29.8, 22.8, 22.7, 21.9, 19.3, 19.2, 13.8, 13.7, 13.5; HRMS (ESI) calcd for C_19_H_21_BrF_3_O_4_S [M − H]^+^ 481.0296, found 481.0305.

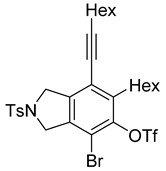

***o*-6f**: This compound was prepared using GPB and isolated in 78% yield after purification by column chromatography. ^1^H-NMR (CDCl_3_, 500 MHz): δ 7.80–7.76 (m, 2H), 7.36–7.33 (m, 2H), 4.71 (s, 2H), 4.60 (s, 2H), 2.87–2.83 (m, 2H), 2.46 (t, 2H, *J* = 7.0 Hz), 2.42 (s, 3H), 1.65–1.42 (m, 6H), 1.38–1.25 (m, 10H), 0.95–0.91 (m, 3H), 0.90–0.86 (m, 3H); ^13^C-NMR (CDCl_3_, 125 MHz): δ 144.1, 144.1, 141.0, 139.7, 136.9, 133.6, 130.0, 127.5, 122.4 (CF_3_), 120.4, 119.8 (CF_3_), 117.3 (CF_3_), 110.5, 101.9, 74.8, 56.0, 55.1, 31.4, 31.3, 30.2, 29.4, 29.3, 28.6, 28.5, 22.6, 22.5, 21.5, 19.7, 14.1, 14.0; HRMS (ESI) calcd for C_30_H_38_BrF_3_NO_5_S_2_ [M + H]^+^ 692.1321, found 692.1309.

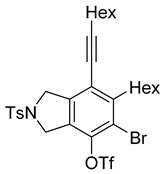

***m*-6f**: This compound was isolated as minor isomer in an inseparable mix with previous compound with 16% yield after purification using column chromatography. ^1^H-NMR (CDCl_3_, 500 MHz): δ 7.78–7.75 (m, 2H), 7.35–7.32 (m, 2H), 4.73 (s, 2H), 4.61 (s, 2H), 2.96–2.91 (m, 2H), 2.48 (t, 2H, *J* = 7.1 Hz), 2.41 (s, 3H), 1.65–1.28 (m, 16H), 0.95–0.86 (m, 6H); HRMS (ESI) calcd for C_30_H_38_BrF_3_NO_5_S_2_ [M + H]^+^ 692.1321, found 692.1306.

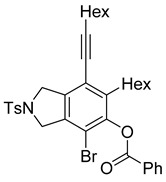

***o*-6f-Br**: This compound was formed by GPB in an inseparable mixture of two isomers (ratio 2:1) with 30% overall yield after column chromatography. ^1^H-NMR (CDCl_3_, 500 MHz): δ (major isomer) 8.22–8.19 (m, 2H), 7.81–7.77 (m, 2H), 7.57–7.51 (m, 3H), 7.37–7.33 (m, 2H), 4.65–4.62 (m, 2H), 4.59–4.54 (m, 2H), 2.56–2.38 (m, 7H), 1.66–1.18 (m, 16H) , 0.96–0.87 (m, 6H); ^13^C-NMR (CDCl_3_, 125 MHz): δ (all discernible signals for both isomers) 164.0, 163.3, 146.4, 145.89, 143.92, 143.84, 139.8, 138.9, 137.4, 135.4, 135.4, 134.5, 134.2, 134.0, 133.64, 130.6, 130.5, 130.3, 130.0, 129.96, 128.9, 128.8, 128.7, 127.6, 127.5, 119.2, 117.8, 111.0, 100.3, 99.6, 75.6, 75.3, 55.8, 55.2, 54.4, 52.7, 35.2, 31.6, 31.4, 31.3, 29.7, 29.5, 29.3, 29.2, 28.9, 28.7, 28.6, 22.6, 22.5, 21.5, 19.7, 19.6, 14.1, 14.0; HRMS (ESI) calcd for C_36_H_43_BrNO_4_S [M + H]^+^ 664.2091, found 664.2079.

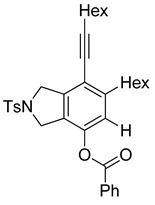

***o*-6f-H**: This compound was isolated in 24.5% using GPB after purification by column chromatography. ^1^H-NMR (CDCl_3_, 500 MHz): δ 8.17–8.13 (m, 2H), 7.78–7.74 (m, 2H), 7.69–7.64 (m, 1H), 7.56–7.50 (m, 2H), 7.34–7.29 (m, 2H), 6.94 (s, 1H), 4.66 (s, 2H), 4.57 (s, 2H), 2.75–2.68 (m, 2H), 2.50–2.44 (m, 2H), 2.41 (s, 3H), 1.66–1.53 (m, 4H), 1.52–1.44 (m, 2H), 1.39–1.24 (m, 10H), 0.97–0.91 (m, 3H), 0.90–0.85 (m, 3H); ^13^C-NMR (CDCl_3_, 125 MHz) δ 164.1, 146.6, 144.4, 143.7, 141.0, 134.0, 133.9, 130.3, 129.9, 128.8, 128.7, 127.6, 125.8, 121.2, 116.4, 98.8, 75.6, 54.5, 52.4, 34.2, 31.7, 31.4, 30.5, 29.7, 29.1, 28.8, 28.6, 22.6, 22.6, 21.5, 19.6, 14.1; HRMS (ESI) calcd for C_36_H_44_NO_4_S [M + H]^+^ 586.2986, found 586.2990.

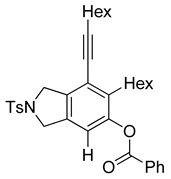

***m*-6f-H**: This compound was prepared using GPB and isolated in 51.5% yield (with 85% purity) after purification by column chromatography. ^1^H-NMR (CDCl_3_, 500 MHz): δ 8.18–8.15 (m, 2H), 7.80–7.76 (m, 2H), 7.67–7.62 (m, 1H), 7.54–7.49 (m, 2H), 7.35–7.30 (m, 2H), 6.88 (s, 1H), 4.64 (s, 2H), 4.62 (s, 2H), 2.69–2.64 (m, 2H), 2.49–2.44 (m, 2H), 2.42 (s, 3H), 1.65–1.57 (m, 2H), 1.54–1.42 (m, 4H), 1.38–1.17 (m, 10 H), 0.94–0.90 (m, 3H), 0.81–0.76 (m, 3H); ^13^C-NMR (CDCl_3_, 125 MHz): δ 165.2, 148.8, 143.7, 136.6, 136.5, 134.0, 133.8, 133.7, 130.1, 129.9, 129.2, 128.7, 127.6, 120.4, 116.1, 99.6, 75.8, 54.2, 54.1, 31.5, 31.4, 29.6, 29.3, 28.8, 28.7, 28.6, 22.6, 22.5, 21.5, 19.6, 14.1, 14.0.

## 4. Conclusions

In conclusion, we developed a formal hydration method of arynes generated from hexadehydro Diels-Alder reaction. While direct use of water does not efficiently trap the *in situ* generated arynes to generate phenolic products, silver trifluroacetate (AgO_2_CCF_3_) can behave as an effective water surrogate in these reactions. This is probably due to the improved miscibility and reactivity of AgO_2_CCF_3_ with arynes, compared to water, to generate the corresponding trifluoroacetoxy organosilver arene intermediates, and, upon treating, with silica gel, these intermediates readily undergo protonolysis of their carbon–silver bonds and hydrolysis of the trifluoroacetyl groups. This protocol can be extended to the synthesis of α-halofunctionalized phenol derivatives by simply adding NBS or NIS to the reaction along with silver trifluroacetate, which provided α-bromo or α-iodophenol derivatives in good yield. Interestingly, the similar reactions with NCS afforded only the corresponding protonated products instead of the expected α-chlorophenols derivatives. Unexpectedly, reactions of substrates containing trialkylsilyl substituents on 1,3-diynes provided α-halotrifluoroacetates rather than their hydrolyzed products. Trapping the same arynes with other oxygen-based nucleophiles containing a silver counter cation, along with NXS, generated α-halooxyfunctionalized products in good yields.

## Supplementary Materials

Supplementary materials can be accessed at: http://www.mdpi.com/1420-3049/20/09/15862/s1.
