# Supplementary material for: Synthesis of Phenolic Compounds by Trapping Arynes with a Hydroxy Surrogate
Source: Molecules. 2015 Aug 31;20(9):15862–80. doi: 10.3390/molecules200915862 (PMC6331853; doi:10.3390/molecules200915862)
Supplement: Supplementary file 1 [file molecules-20-15862-s001.pdf]

Supplementary Materials

Contents

General Information S1

Preparation of the Substrates S1–S3

^{1}H-, ^{13}C-, and ^{19}F-NMR Spectra of the Products S3–S28

Mulliken Population Analysis S28

1. General Information

Reactions were carried out in oven-dried glassware unless otherwise noted. Compounds were purchased from Aldrich or Acros or TCI America or Oakwood Chemicals unless otherwise noted. Toluene, acetonitrile and dichloromethane were distilled over calcium hydride (CaH_2) under nitrogen atmosphere. THF was distilled over sodium-benzophenone ketyl under nitrogen atmosphere. Column chromatography was performed using silica gel 60 Å (32–63 mesh) purchased from Silicycle Inc. Analytical thin layer chromatography (TLC) was performed on 0.25 mm E. Merck precoated silica gel 60 (particle size 0.040−0.063 mm). ^{1}H-NMR and ^{13}C-NMR spectra were recorded on a Bruker AV-500 spectrometer. ^{19}F-NMR spectrum was recorded in Varian Mercury-Vx-300 spectrometer.

2. Preparation of Substrates

1,3-*bis*-Diynes were prepared following the multistep procedure described below.

2.1. Symmetrical 1,3-bis-Diynes

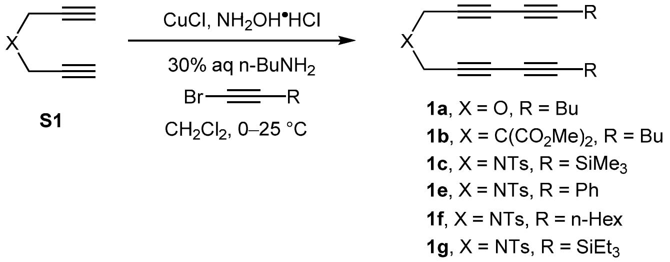

General Procedure for the Cadiot–Chodkiewicz Reaction

In a two-neck round-bottom flask containing CuCl (1.2 mmol, 0.6 equiv.) was added 30% aqueous *n*-$BuNH_2$ solution (4 mL/mmol of diyne) under nitrogen flow. A pinch of NH_2OH•HCl was added until a blue color disappeared. A solution of diyne (2.0 mmol, 1 equiv.) in dichloromethane was added at 0 °C to the flask and the solution became yellow. A solution of alkynyl bromide (5.0 mmol, 2.5 equiv.) in dichloromethane was added drop wise at 0 °C. The reaction mixture was stirred at room temperature until completion of the reaction monitored by TLC. The nitrogen flow was removed and the biphasic reaction mixture was transferred to a separatory funnel. Dichloromethane layer was separated and dried over anhydrous $MgSO_4$. After filtration, the organic layer was concentrated and the crude material was purified by column chromatography to get pure tetraynes.

2.2. Unsymmetrical 1,3-bis-diyne 1d

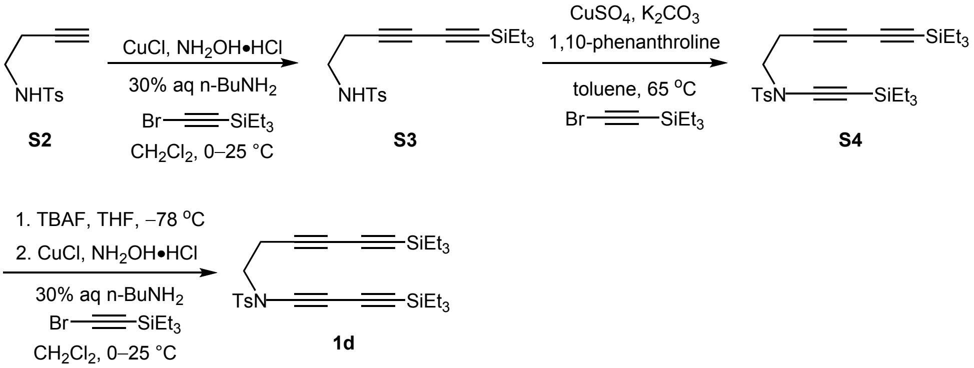

2.2.1. Procedure for the Synthesis of S3

In a two-neck round-bottomed flask containing CuCl (0.6 mmol, 0.3 equiv) was added 30% aqueous *n*-$BuNH_2$ solution (2 mL/mmol of **S2**) under nitrogen flow. A pinch of NH_2OH•HCl was added until a blue color disappeared. A solution of the terminal alkyne **S2** (2.0 mmol, 1.0 equiv.) in dichloromethane was added at 0 °C to the flask and the solution became yellow. A solution of alkynyl bromide (2.4 mmol, 1.2 equiv.) in dichloromethane was added drop wise at 0 °C. The reaction mixture was stirred at room temperature until completion of the reaction monitored by TLC. The nitrogen flow was removed and the biphasic reaction mixture was transferred to a separatory funnel. Dichloromethane layer was separated and dried over anhydrous $MgSO_4$. After filtration, the organic layer was concentrated and the crude material was purified by column chromatography (Hex–EtOAc, 10:1 to 5:1) to get pure **S3** (90% yield).

2.2.2. Procedure for the Synthesis of S4

A mixture of **S3** (1.5 mmol, 1.0 equiv.), $CuSO_4$•$5H_2O$ (0.15 mmol, 10 mol %), 1,10-phenanthroline (0.3 mmol, 20 mol %), K_2CO_3 (3.0 mmol, 2.0 equiv.) and (bromoethynyl)triethylsilane (2.3 mmol, 1.5 equiv.) in dry toluene (10 mL) was stirred at 65 °C for overnight. After completion, the reaction mixture was cooled down to room temperature and filtered through a small pad of silica gel. The filtrate was concentrated under reduced pressure and the crude was purified by column chromatography (Hex–EtOAc, 20:1 to 10:1) to get pure **S4** (82% yield).

2.2.3. Procedure for the Synthesis of 1d

To a stirred solution of **S4** (1.0 mmol, 1 equiv.) in THF at −78 °C, was added TBAF (1.1 mmol, 1.1 equiv.) slowly. After completion of the reaction (usually within a couple of minutes, monitored by TLC) the reaction mixture was diluted with ethyl acetate, transferred in a separatory funnel, washed successively with water and brine. The organic layer was separated, dried over anhydrous $MgSO_4$, filtered, concentrated under reduced pressure and the crude triyne with terminal alkyne was used for the next step.

In a two-neck round-bottomed flask containing CuCl (0.3 mmol, 0.3 equiv.) was added 30% aqueous *n*-$BuNH_2$ solution (2 mL/mmol of the terminal alkyne) under nitrogen flow. A pinch of NH_2OH•HCl was added until a blue color disappeared. A solution of the crude alkyne (roughly 1.0 mmol) in

dichloromethane was added at 0 °C to the flask and the solution became yellow. A solution of alkynyl bromide (1.2 mmol, 1.2 equiv.) in dichloromethane was added drop wise at 0 °C. The reaction mixture was stirred until completion of the reaction monitored by TLC. The nitrogen flow was removed and the biphasic reaction mixture was transferred to a separatory funnel. Dichloromethane layer was separated and dried over anhydrous $MgSO_4$. After filtration, the organic layer was concentrated and the crude material was purified by column chromatography (Hex–EtOAc, 20:1 to 10:1) to get pure **1d** (88% yield, 2 steps).

NMR Spectra of the Products

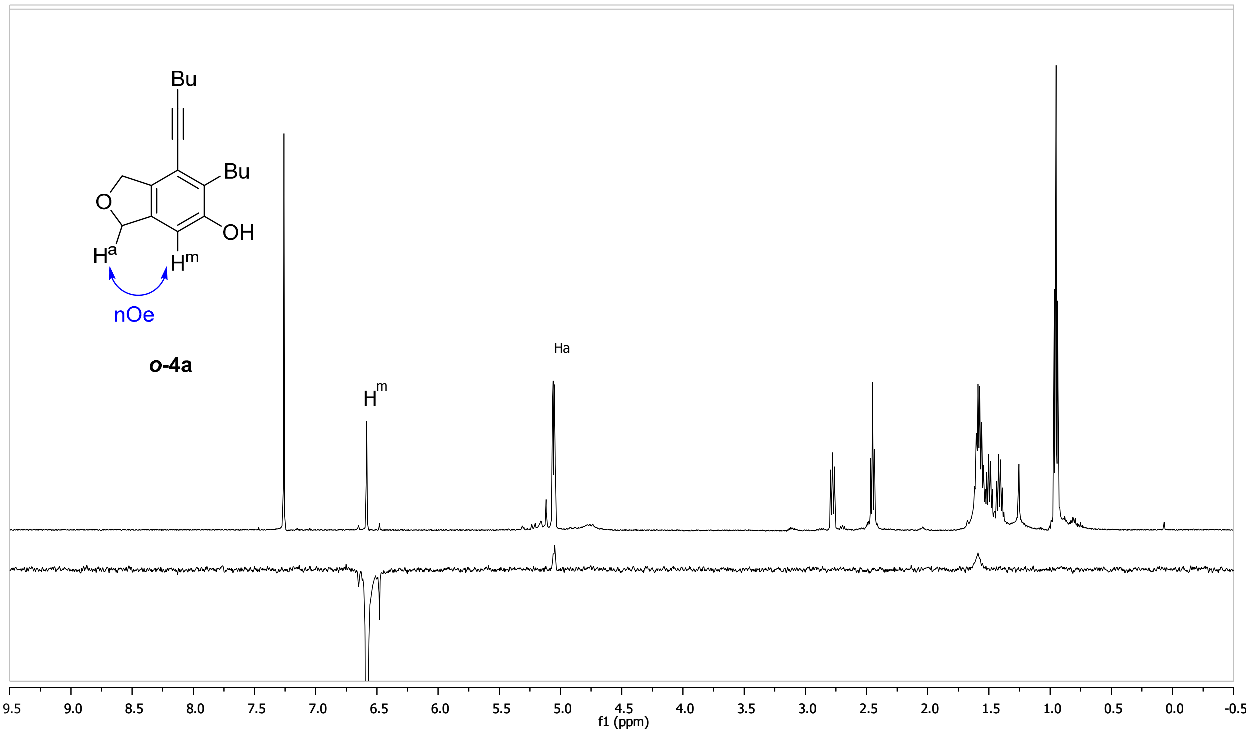

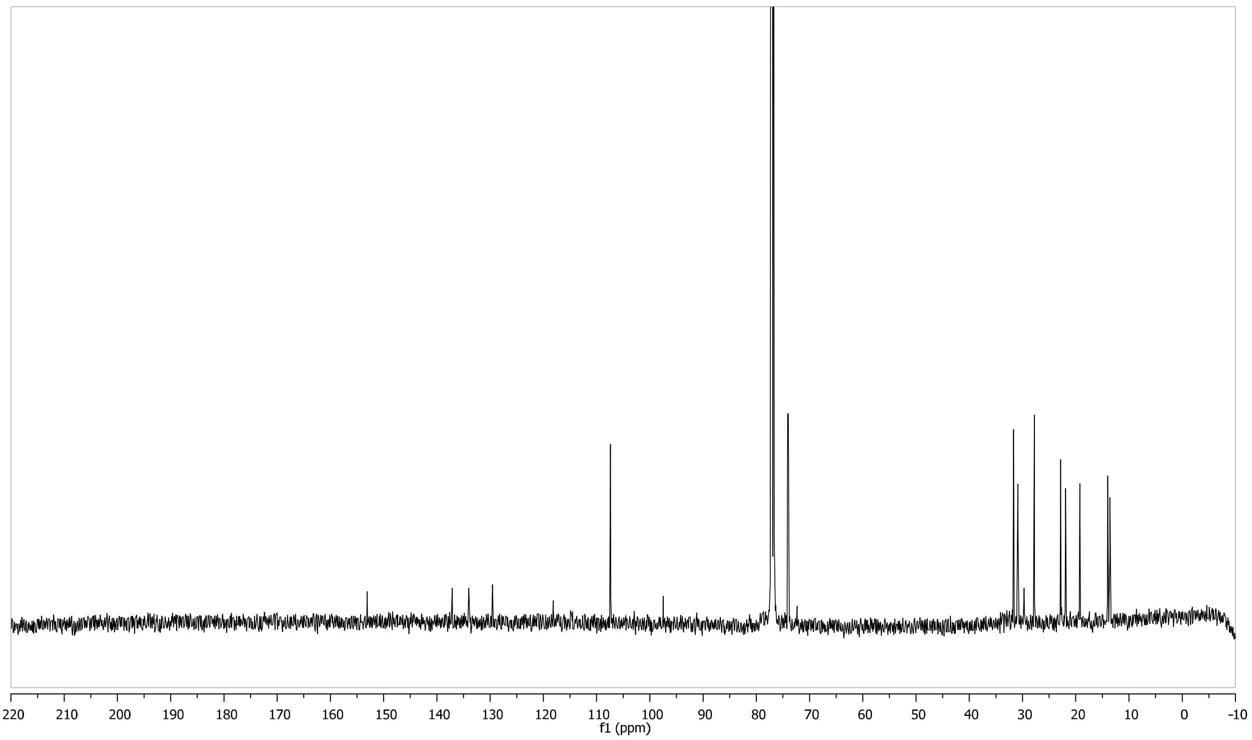
220
210
200
190
180
170
160
150
140
130
120
110
100
90
80
70
60
50
40
30
20
10
0
-10
f1 (ppm)

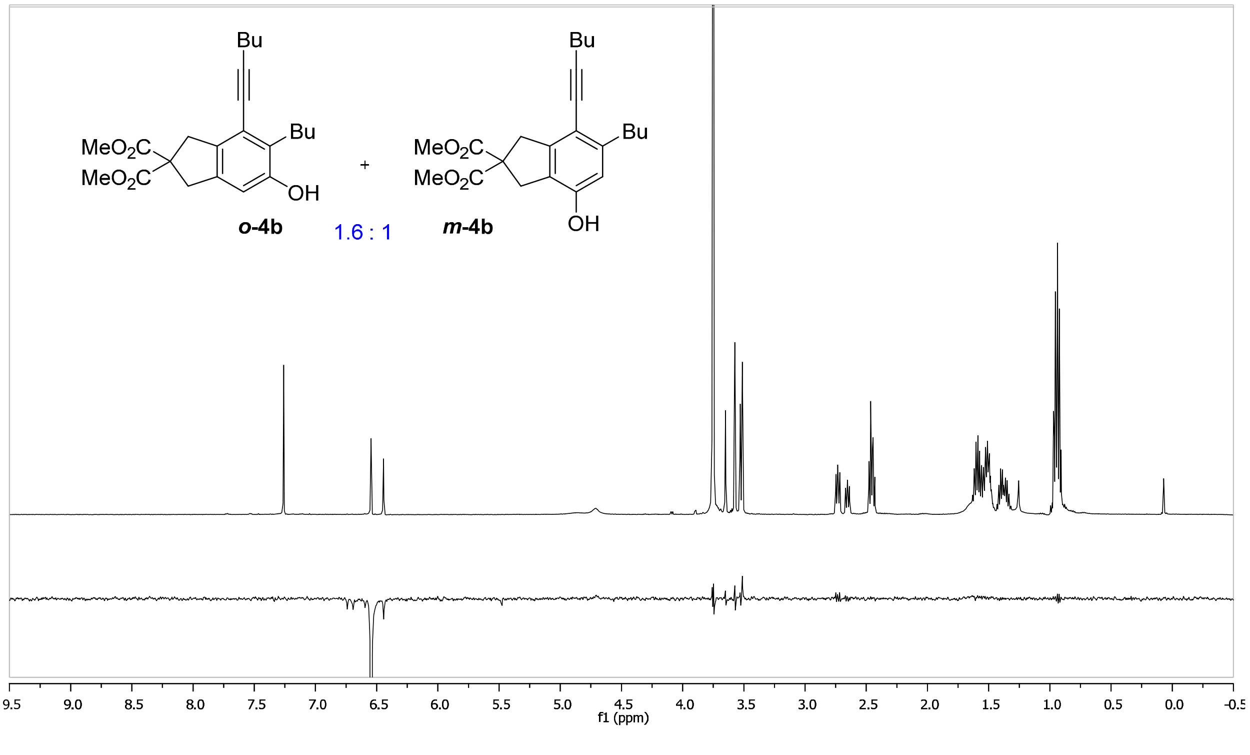
Bu
MeO2C
MeO2C
Bu
OH
o-4b
+
1.6 : 1
Bu
MeO2C
MeO2C
Bu
OH
m-4b
9.5
9.0
8.5
8.0
7.5
7.0
6.5
6.0
5.5
5.0
4.5
4.0
3.5
3.0
2.5
2.0
1.5
1.0
0.5
0.0
-0.5
f1 (ppm)

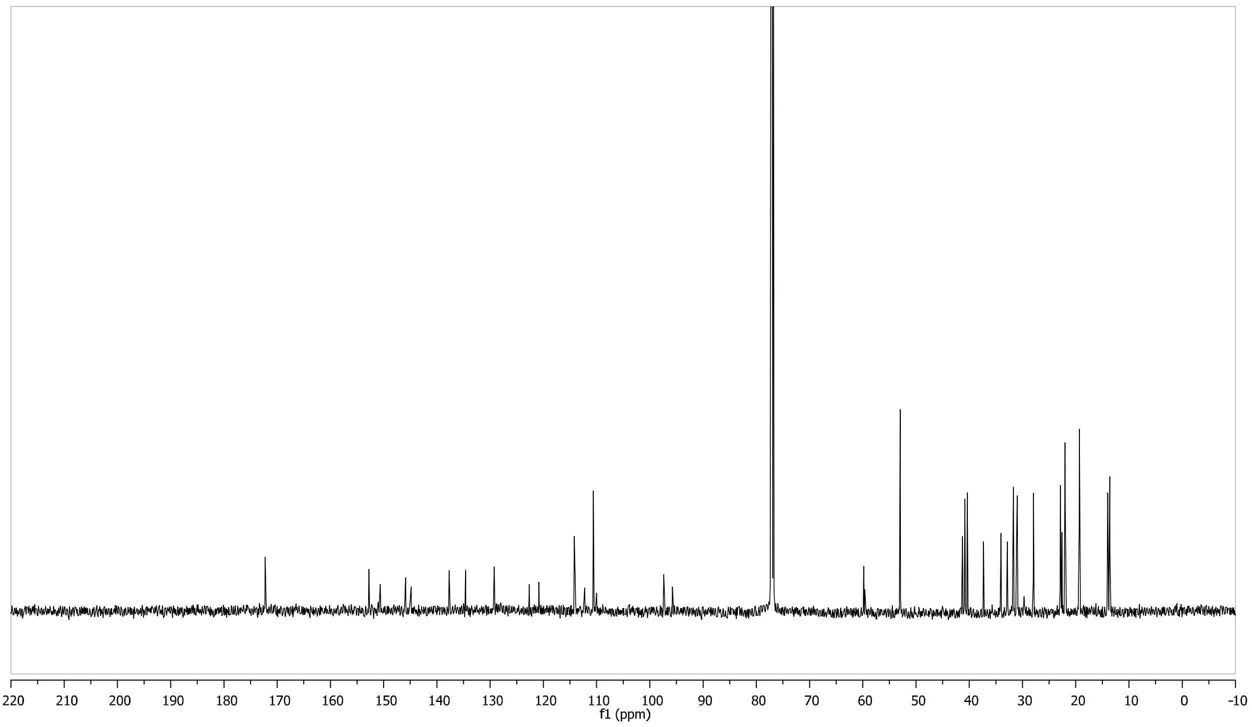
220
210
200
190
180
170
160
150
140
130
120
110
100
90
80
70
60
50
40
30
20
10
0
-10
f1 (ppm)

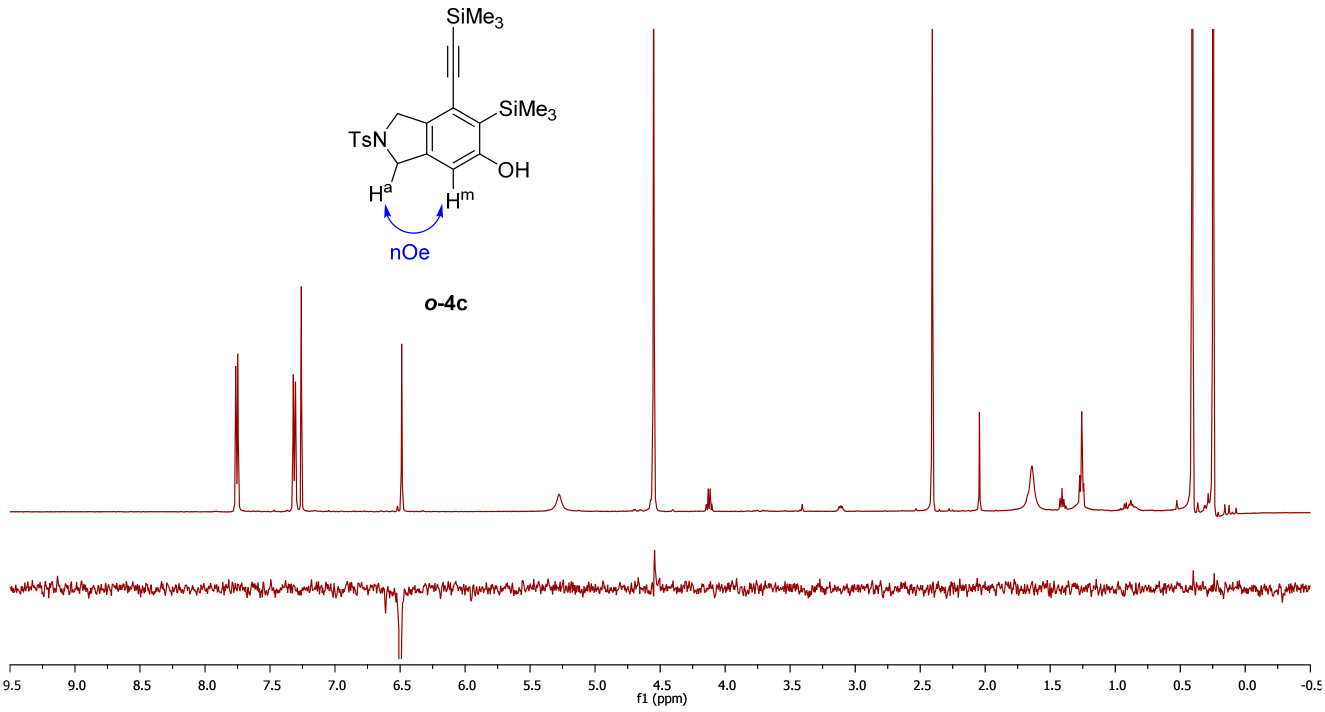
SiMe3
SiMe3
TsN
OH
Ha
Hm
nOe
o-4c
9.5
9.0
8.5
8.0
7.5
7.0
6.5
6.0
5.5
5.0
4.5
4.0
3.5
3.0
2.5
2.0
1.5
1.0
0.5
0.0
f1 (ppm)

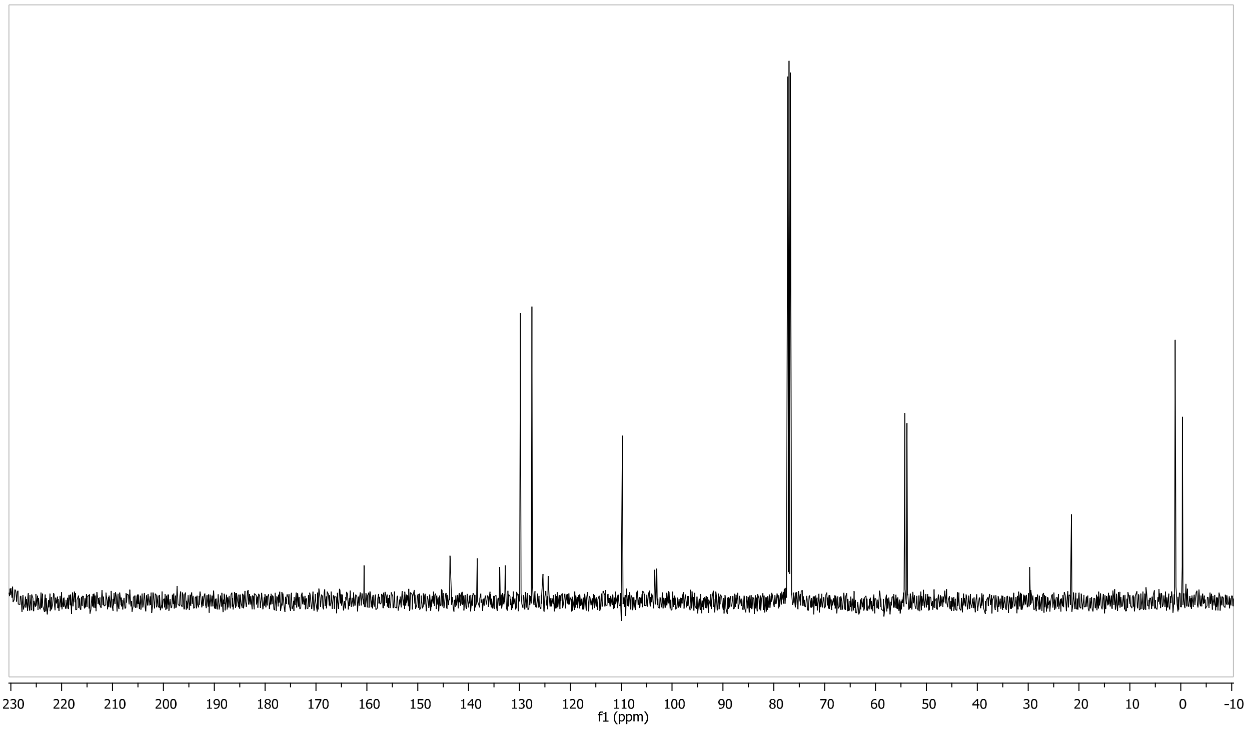

230 220 210 200 190 180 170 160 150 140 130 120 110 100 90 80 70 60 50 40 30 20 10 0 -10
f1 (ppm)

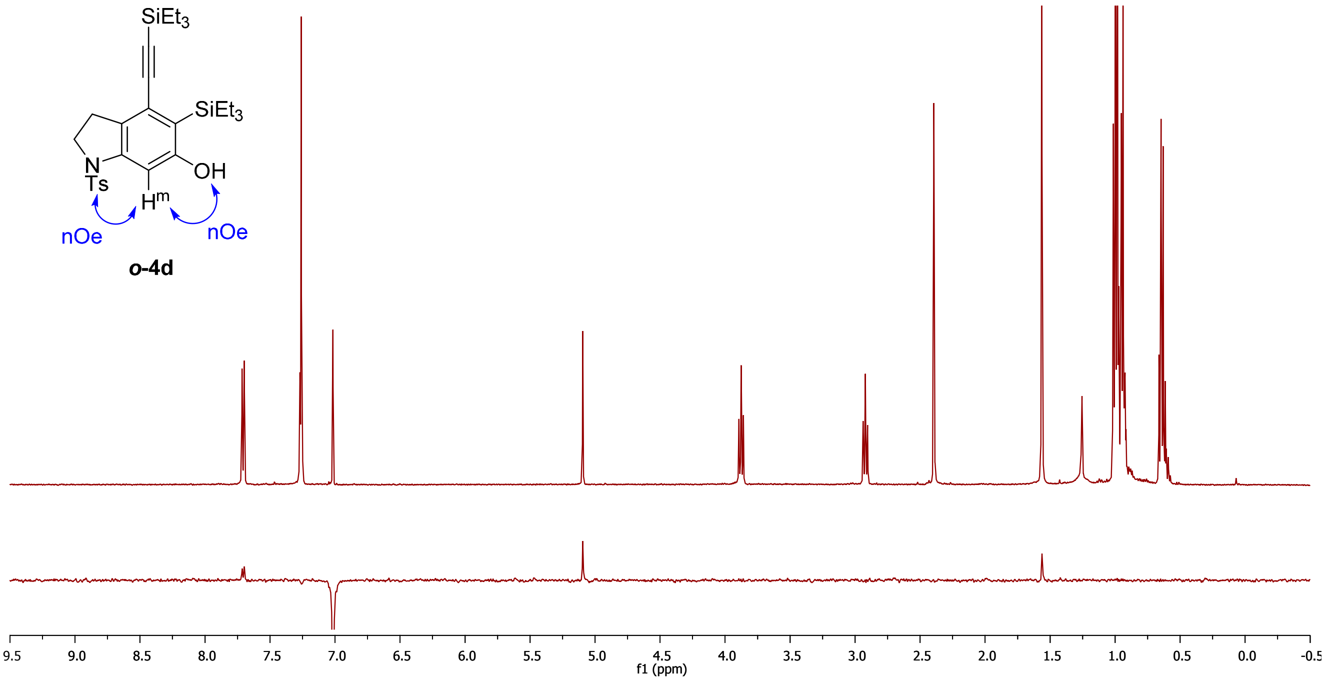

SiEt3
SiEt3
OH
N
Ts
Hm
nOe
nOe
o-4d
9.5 9.0 8.5 8.0 7.5 7.0 6.5 6.0 5.5 5.0 4.5 4.0 3.5 3.0 2.5 2.0 1.5 1.0 0.5 0.0 -0.5
f1 (ppm)

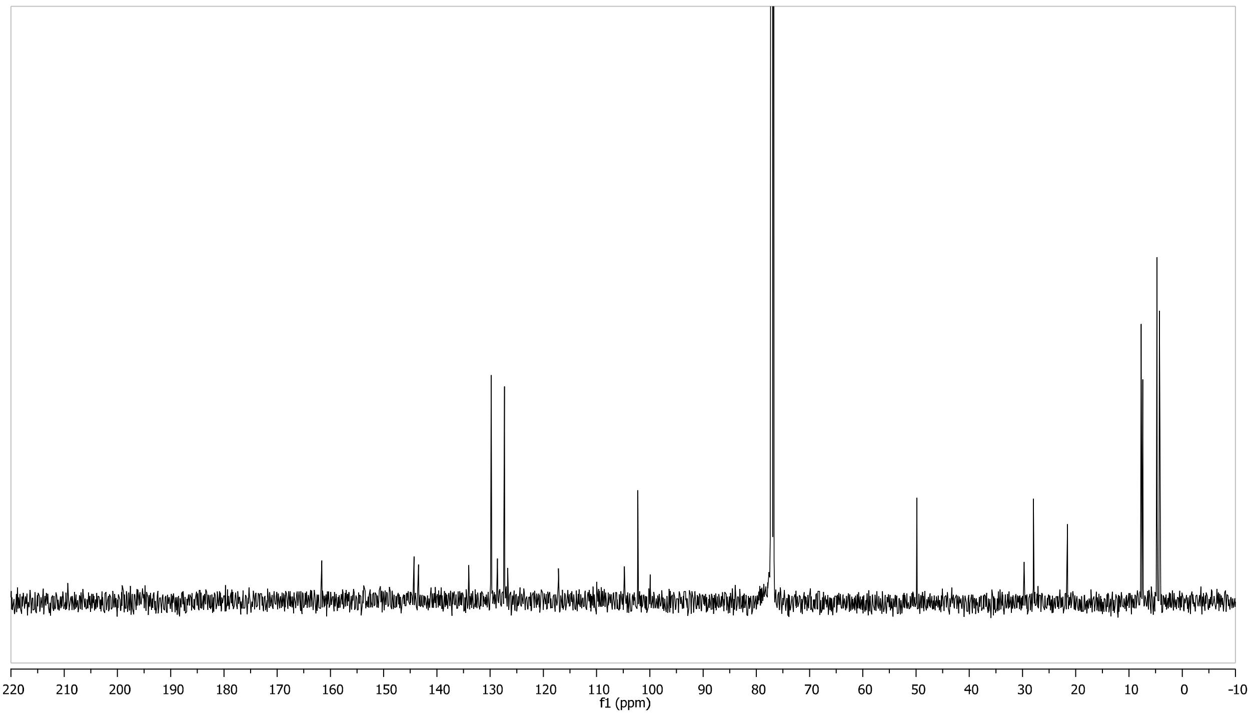
220
210
200
190
180
170
160
150
140
130
120
110
100
90
80
70
60
50
40
30
20
10
0
-10
f1 (ppm)

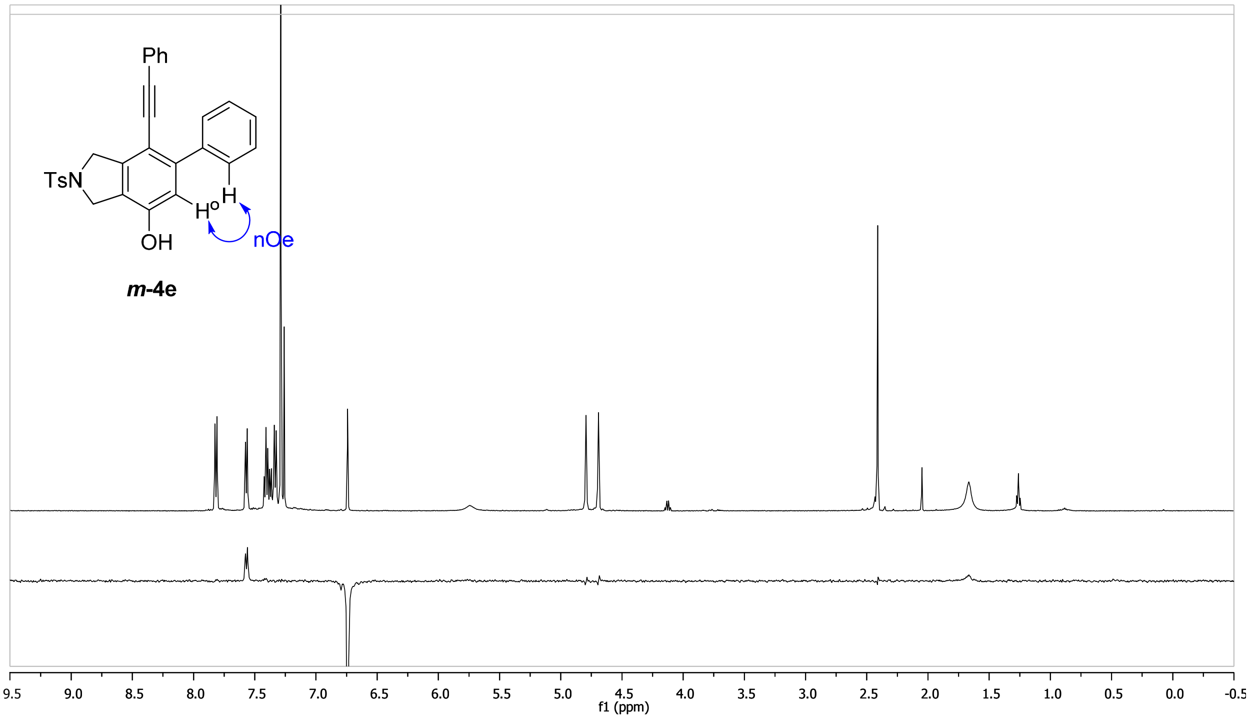
Ph
TsN
H
H
OH
nOe
m-4e
9.5
9.0
8.5
8.0
7.5
7.0
6.5
6.0
5.5
5.0
4.5
4.0
3.5
3.0
2.5
2.0
1.5
1.0
0.5
0.0
-0.5
f1 (ppm)

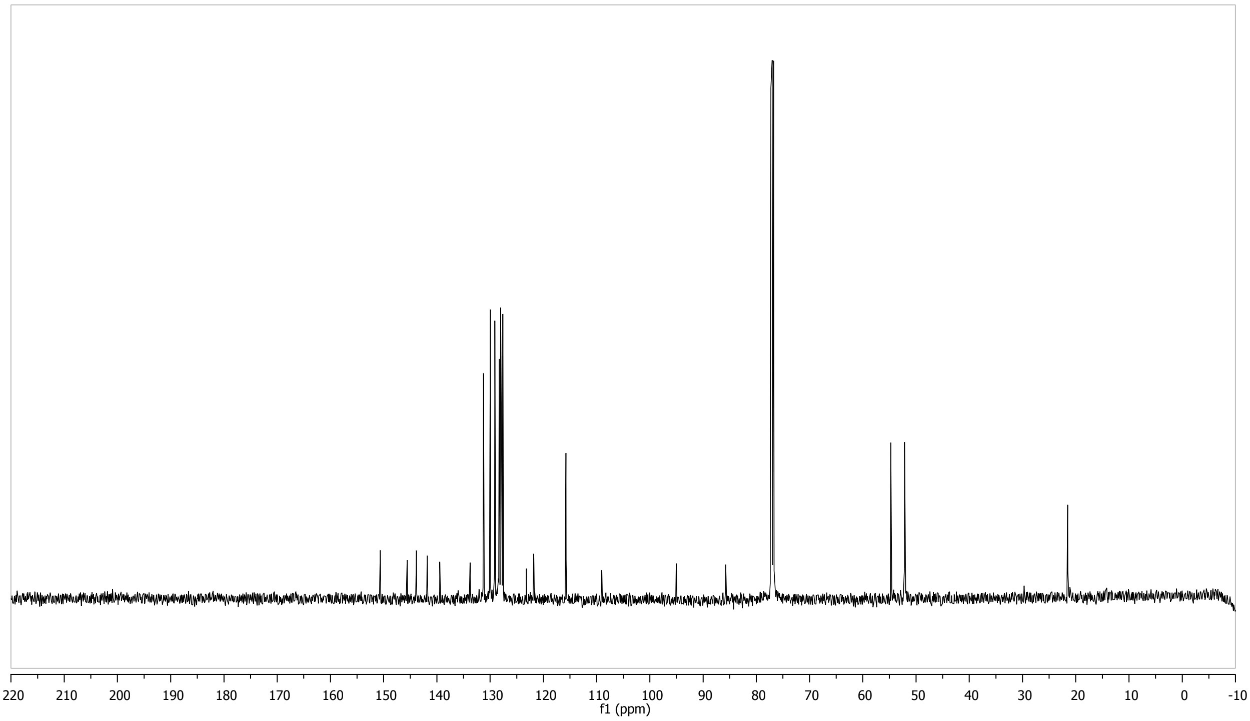
220 210 200 190 180 170 160 150 140 130 120 110 100 90 80 70 60 50 40 30 20 10 0 -10
f1 (ppm)

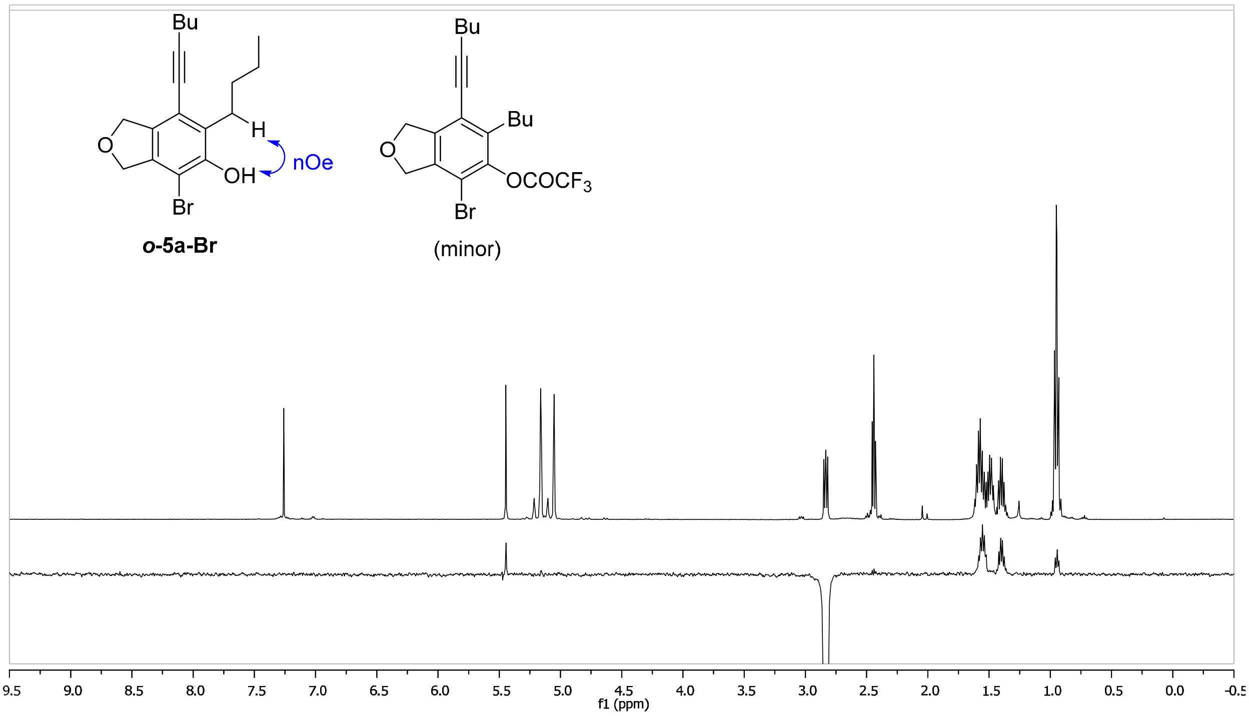
Bu
H
nOe
OH
O
Br
o-5a-Br
Bu
Bu
O
OCOCF3
Br
(minor)
9.5 9.0 8.5 8.0 7.5 7.0 6.5 6.0 5.5 5.0 4.5 4.0 3.5 3.0 2.5 2.0 1.5 1.0 0.5 0.0
f1 (ppm)

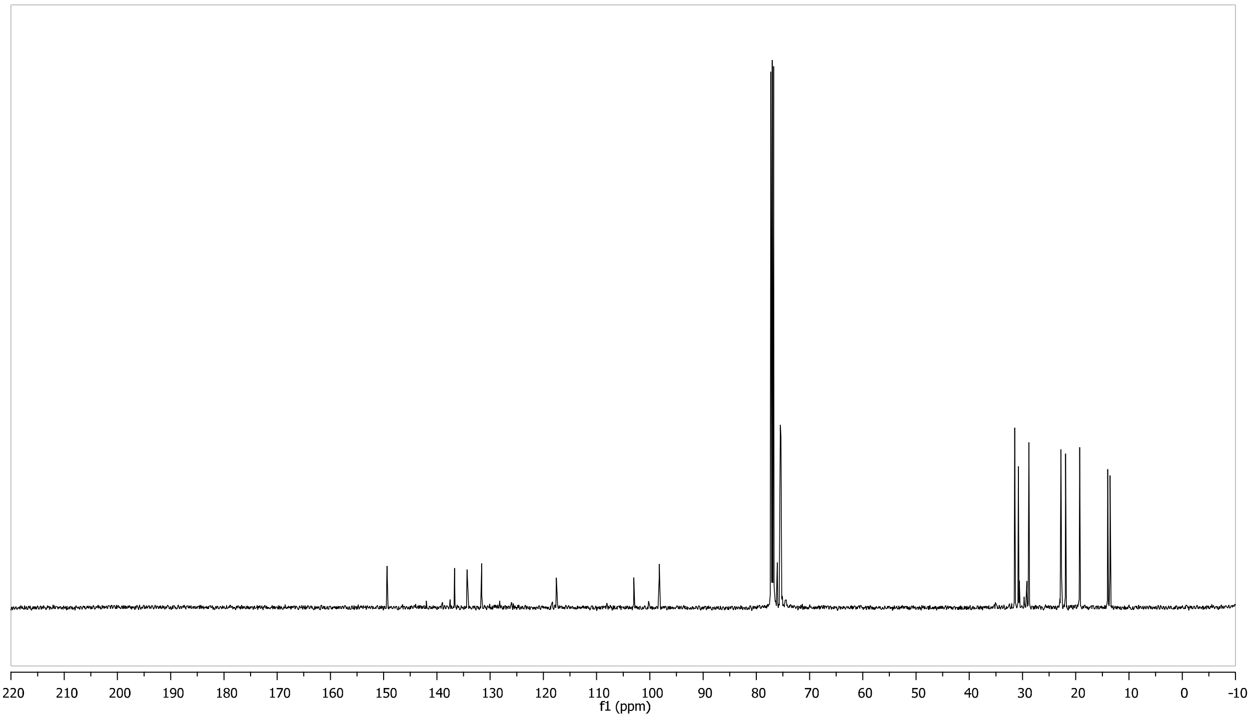
f1 (ppm)

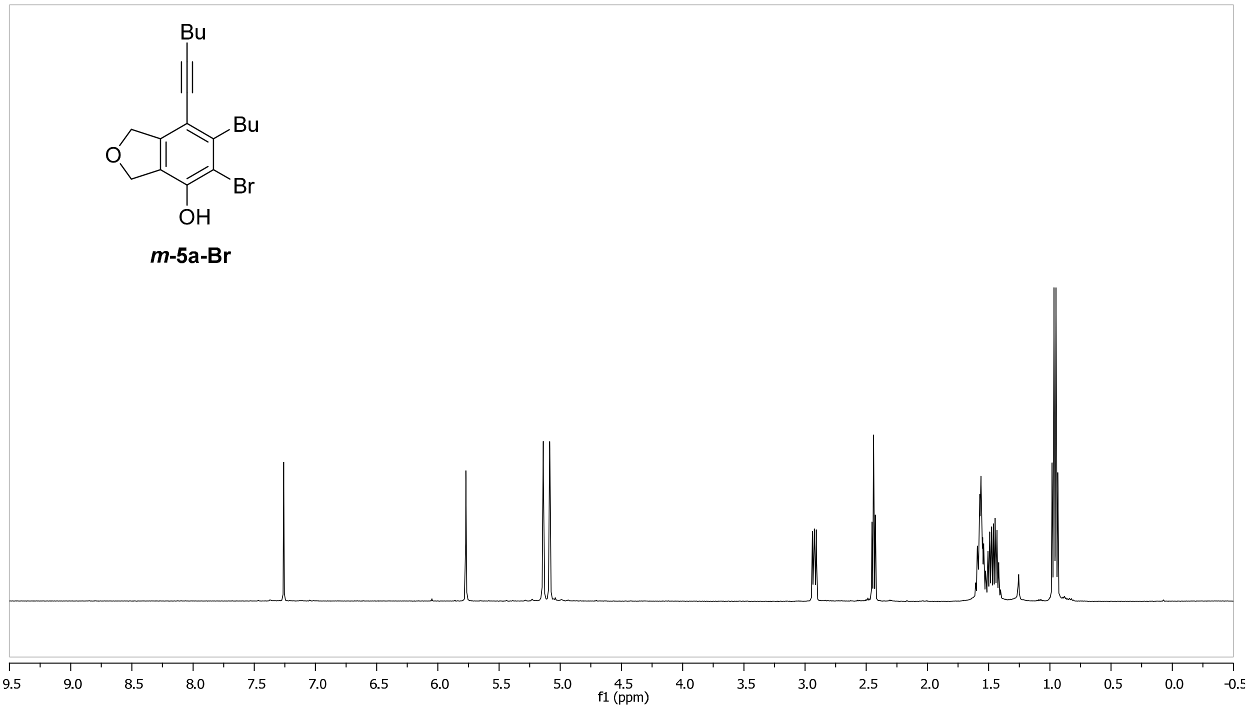
Bu
Bu
O
Br
OH
m-5a-Br
f1 (ppm)

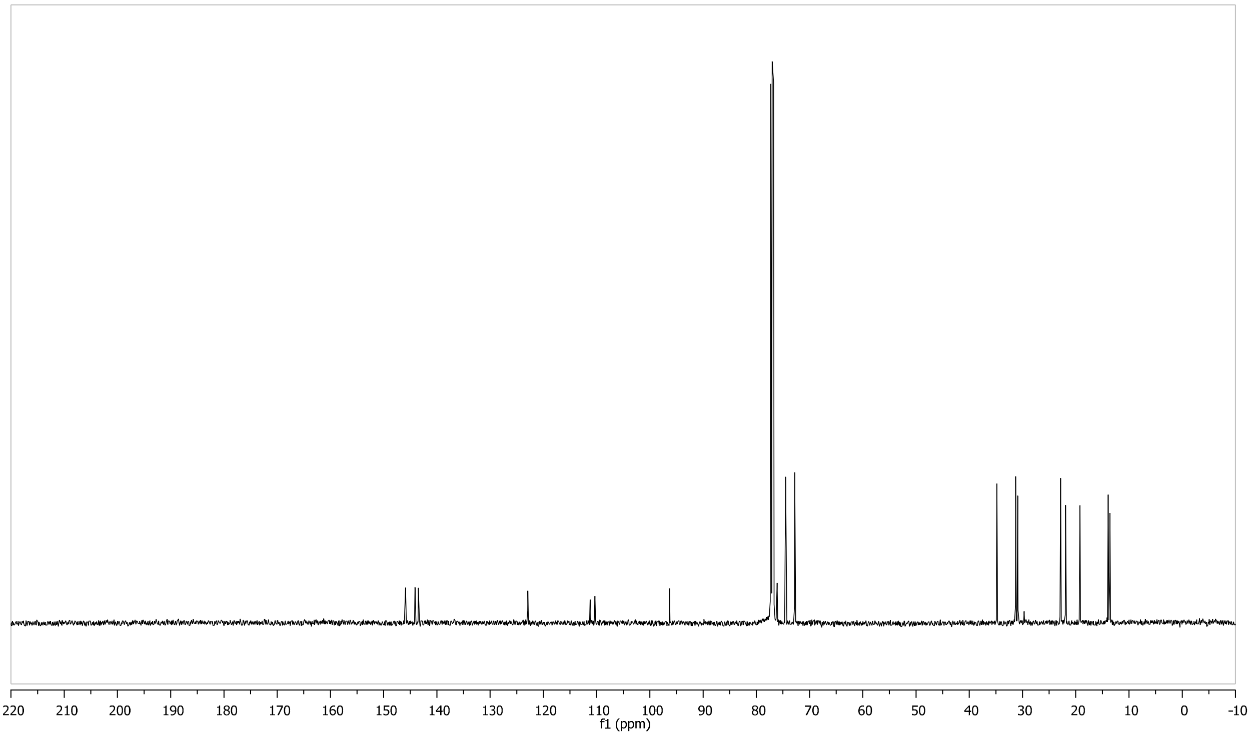

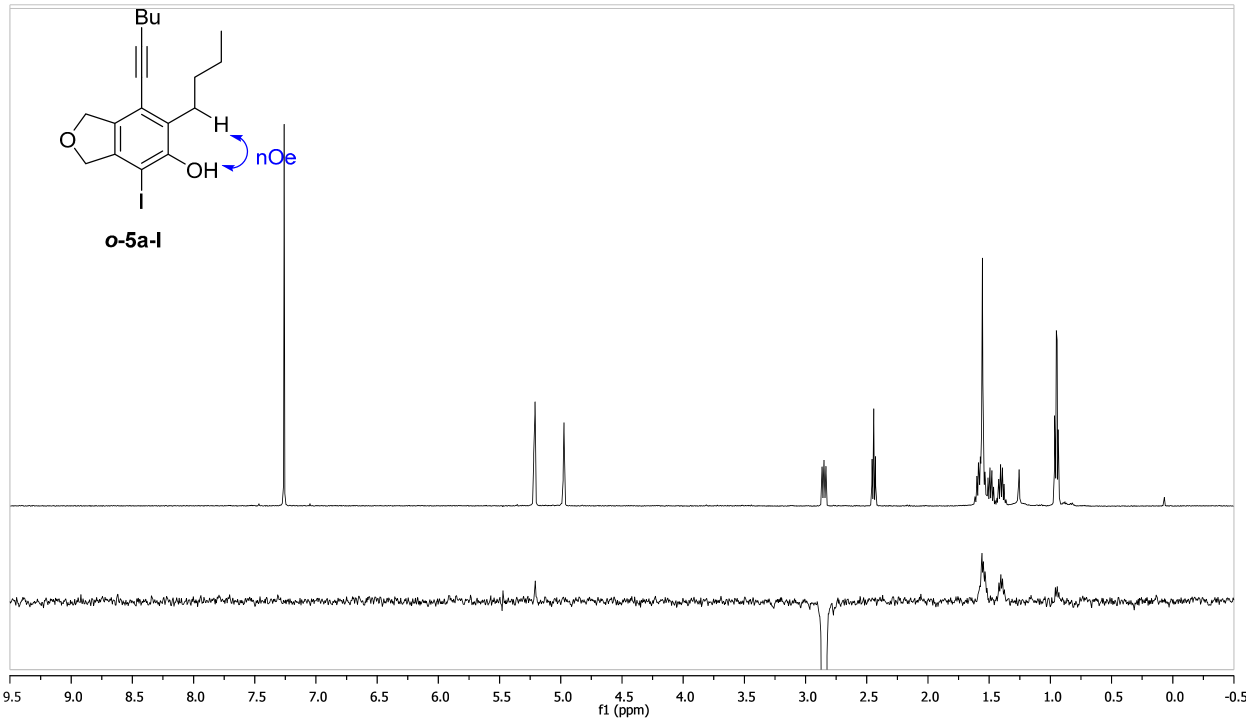

***o*-5a-I**

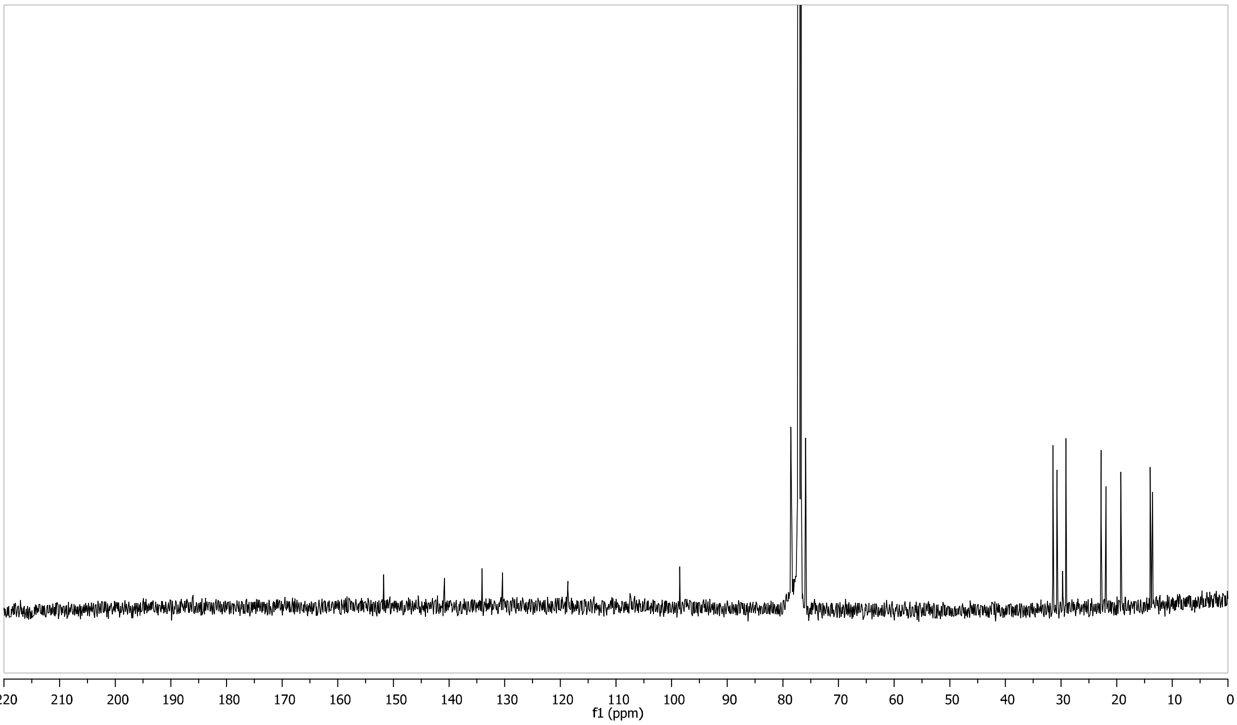
220
210
200
190
180
170
160
150
140
130
120
110
100
90
80
70
60
50
40
30
20
10
0
f1 (ppm)

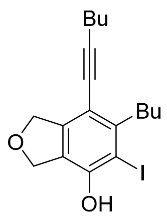
Bu
Bu
O
I
OH

***m*-5a-I**

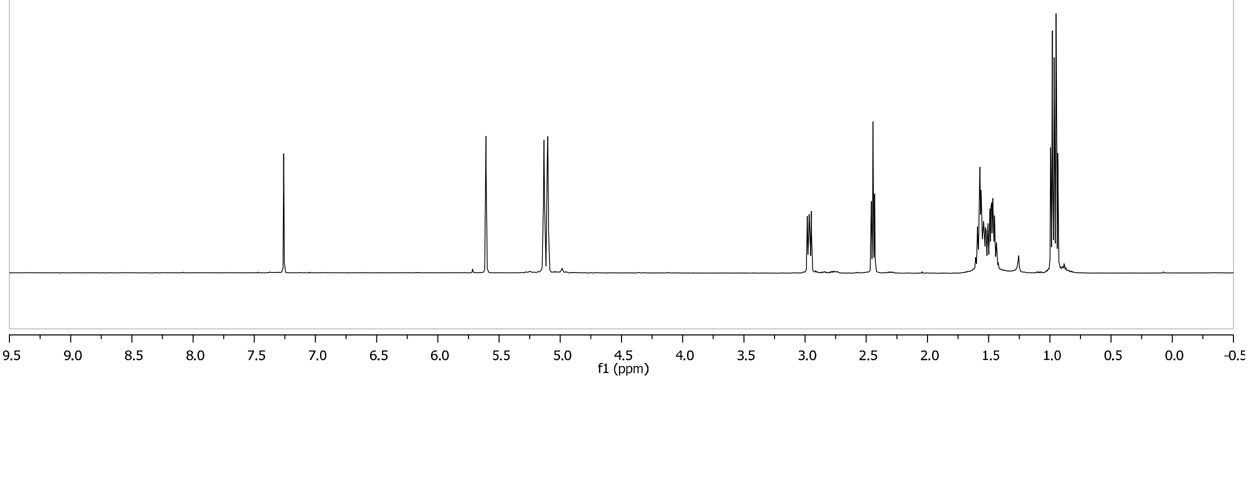
9.5
9.0
8.5
8.0
7.5
7.0
6.5
6.0
5.5
5.0
4.5
4.0
3.5
3.0
2.5
2.0
1.5
1.0
0.5
0.0
f1 (ppm)

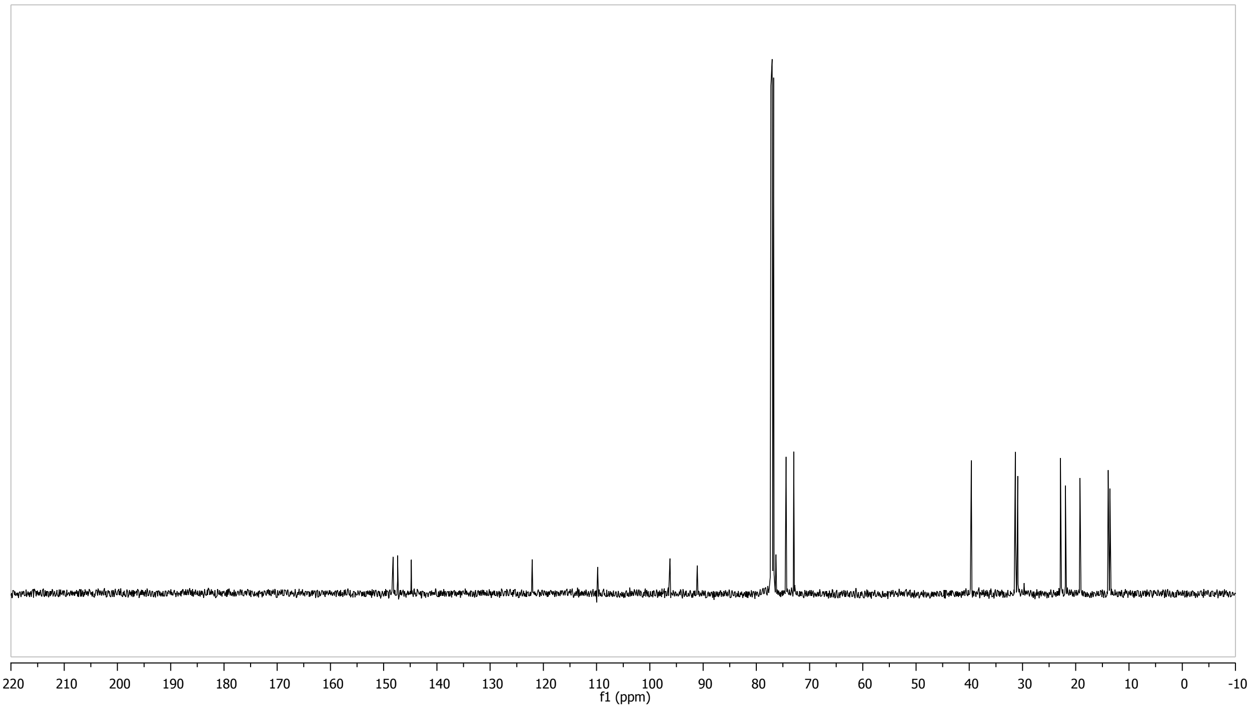
220
210
200
190
180
170
160
150
140
130
120
110
100
90
80
70
60
50
40
30
20
10
0
-10
f1 (ppm)

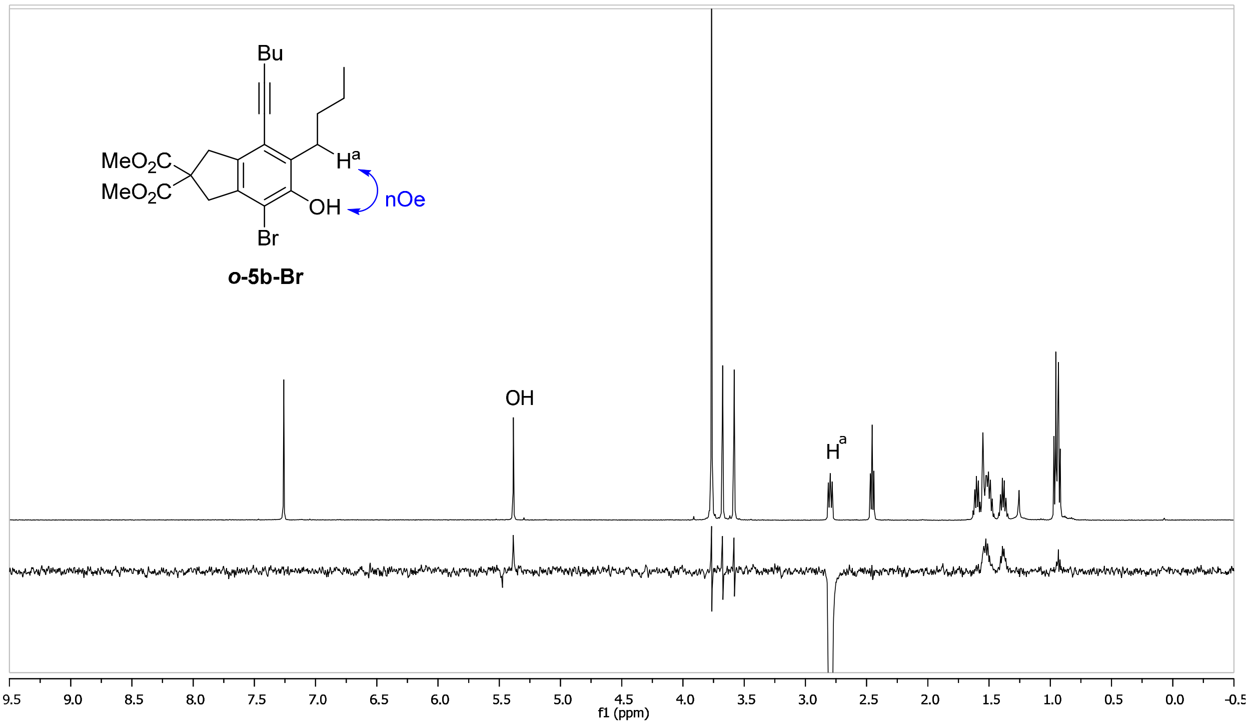
Bu
MeO2C
MeO2C
Ha
OH
nOe
Br
o-5b-Br
OH
Ha
9.5
9.0
8.5
8.0
7.5
7.0
6.5
6.0
5.5
5.0
4.5
4.0
3.5
3.0
2.5
2.0
1.5
1.0
0.5
0.0
f1 (ppm)

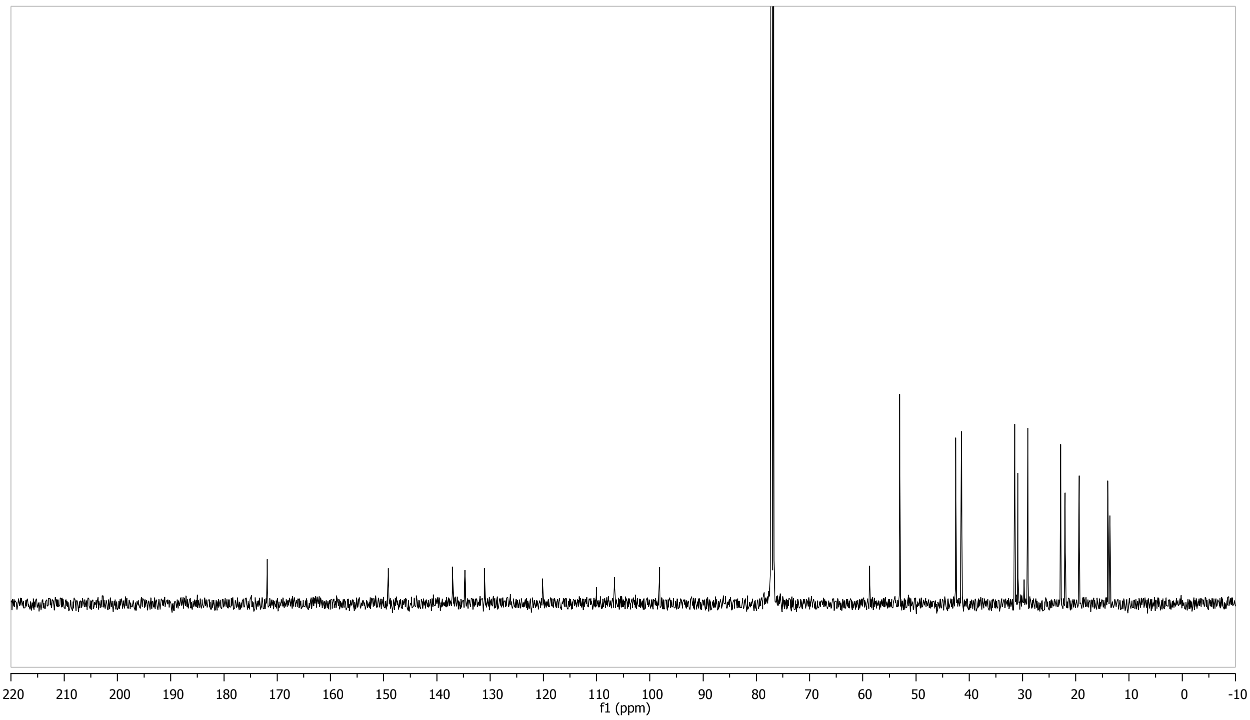
f1 (ppm)

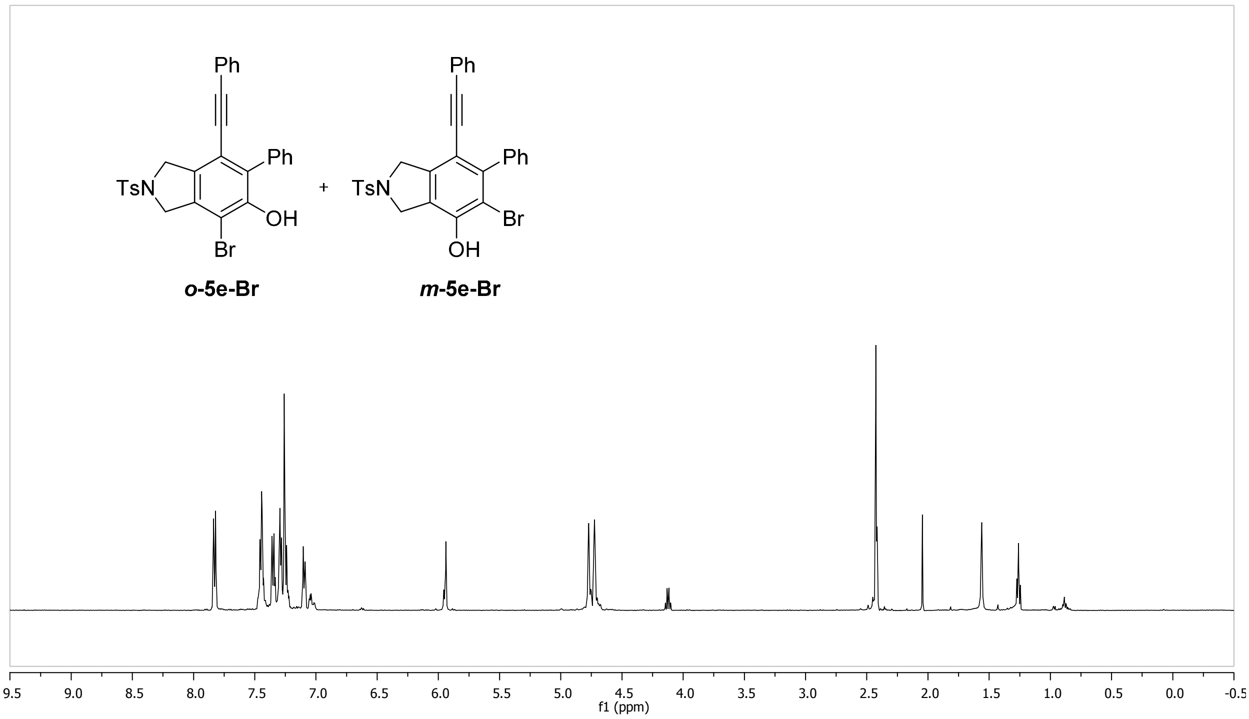
Ph
Ph
TsN
OH
Br
o-5e-Br
+
Ph
Ph
TsN
Br
OH
m-5e-Br
f1 (ppm)

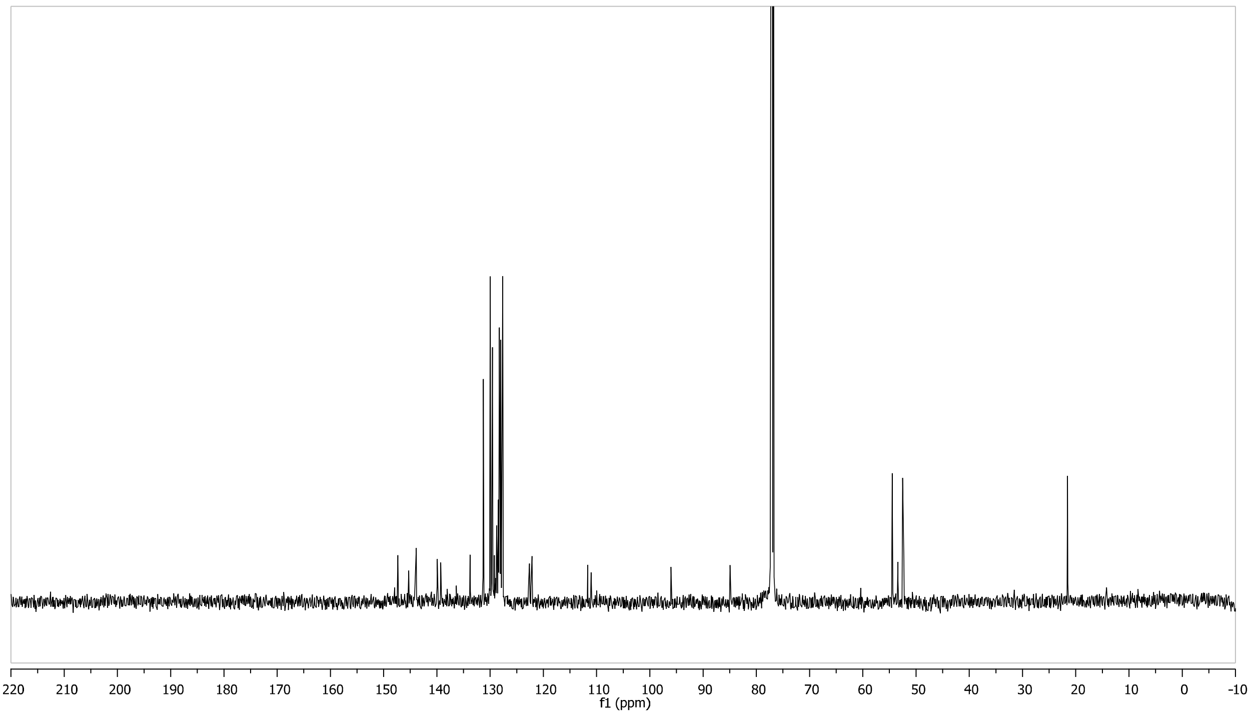
220
210
200
190
180
170
160
150
140
130
120
110
100
90
80
70
60
50
40
30
20
10
0
-10
f1 (ppm)

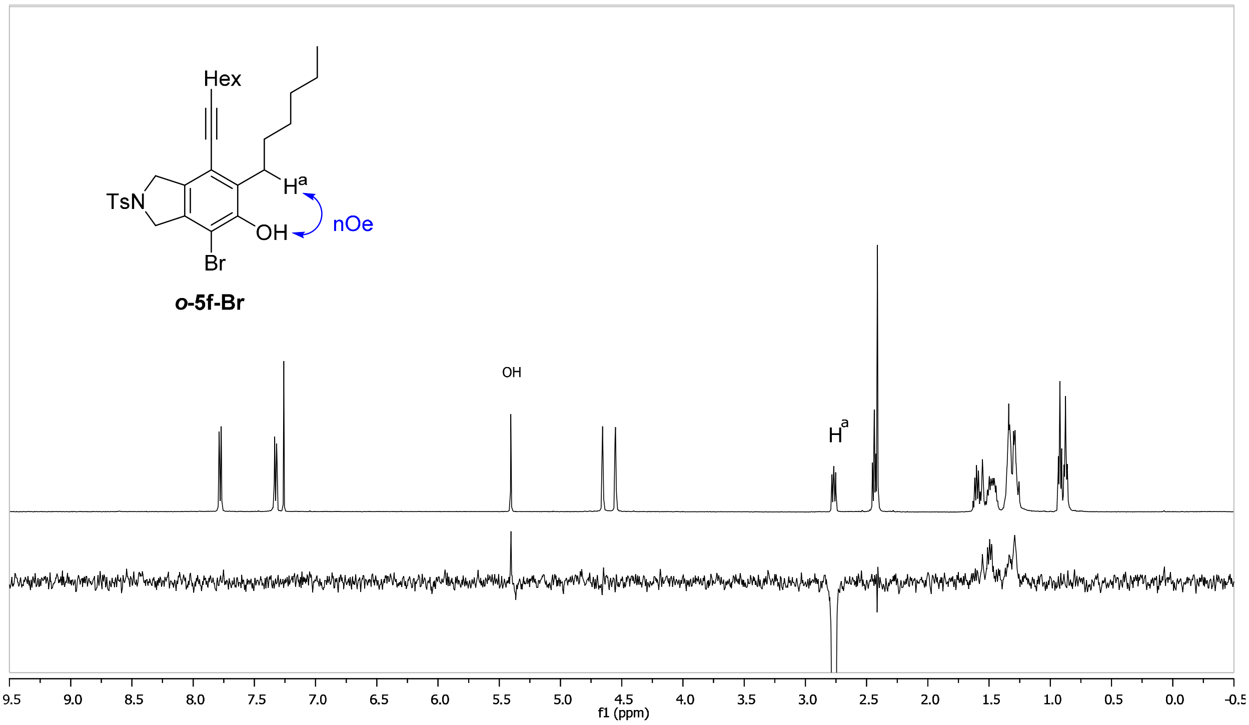
Hex
TsN
H^a
nOe
OH
Br
o-5f-Br
OH
H^a
9.5
9.0
8.5
8.0
7.5
7.0
6.5
6.0
5.5
5.0
4.5
4.0
3.5
3.0
2.5
2.0
1.5
1.0
0.5
0.0
f1 (ppm)

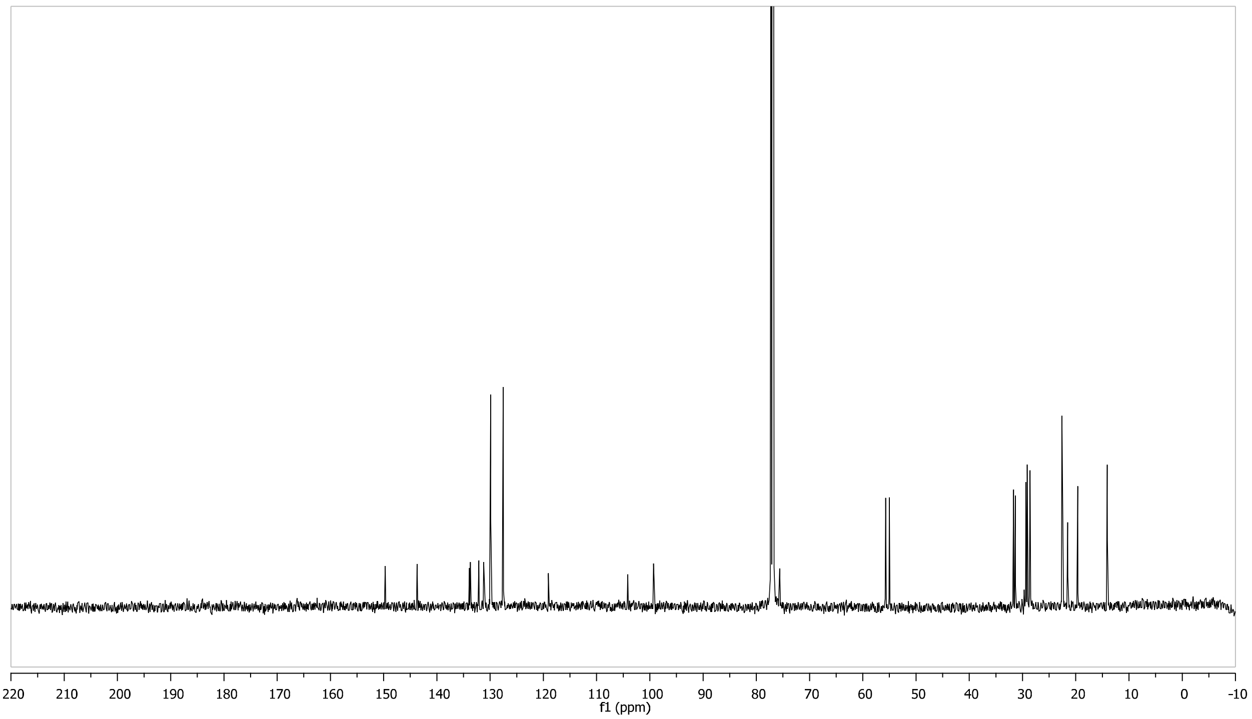
220
210
200
190
180
170
160
150
140
130
120
110
100
90
80
70
60
50
40
30
20
10
0
-10
f1 (ppm)

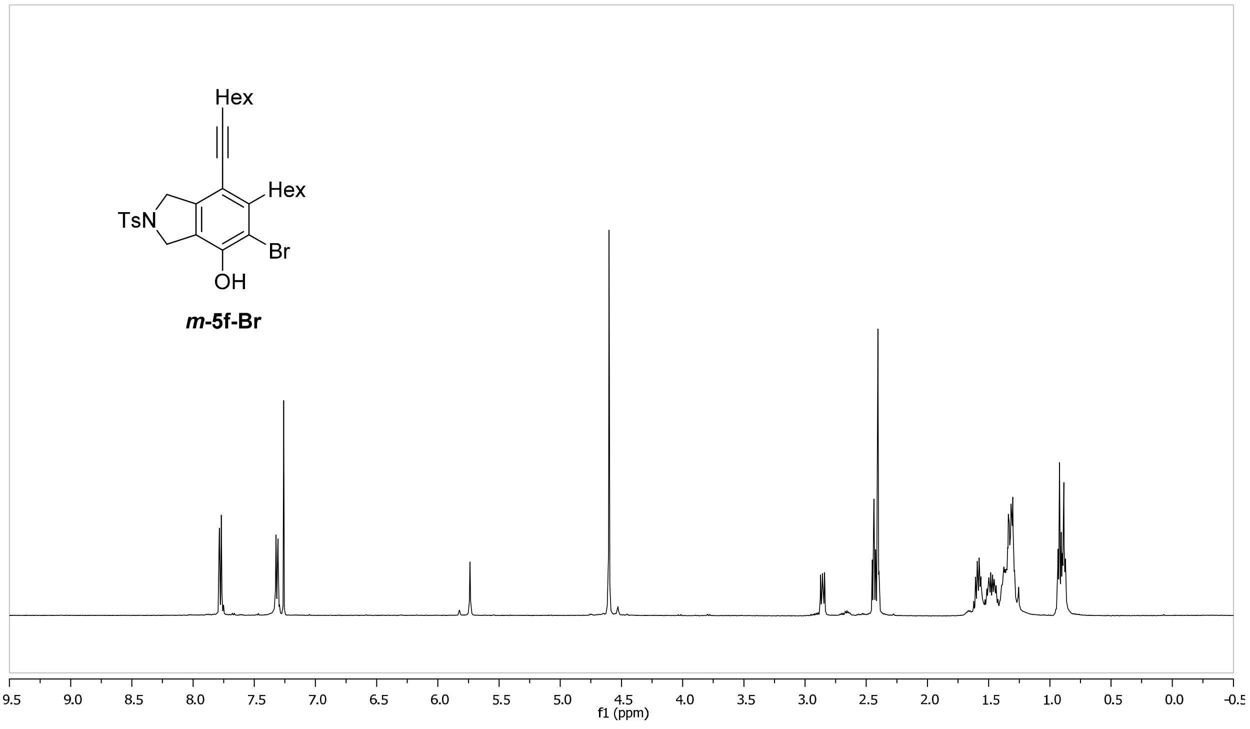
Hex
Hex
TsN
Br
OH
m-5f-Br
9.5
9.0
8.5
8.0
7.5
7.0
6.5
6.0
5.5
5.0
4.5
4.0
3.5
3.0
2.5
2.0
1.5
1.0
0.5
0.0
-0.5
f1 (ppm)

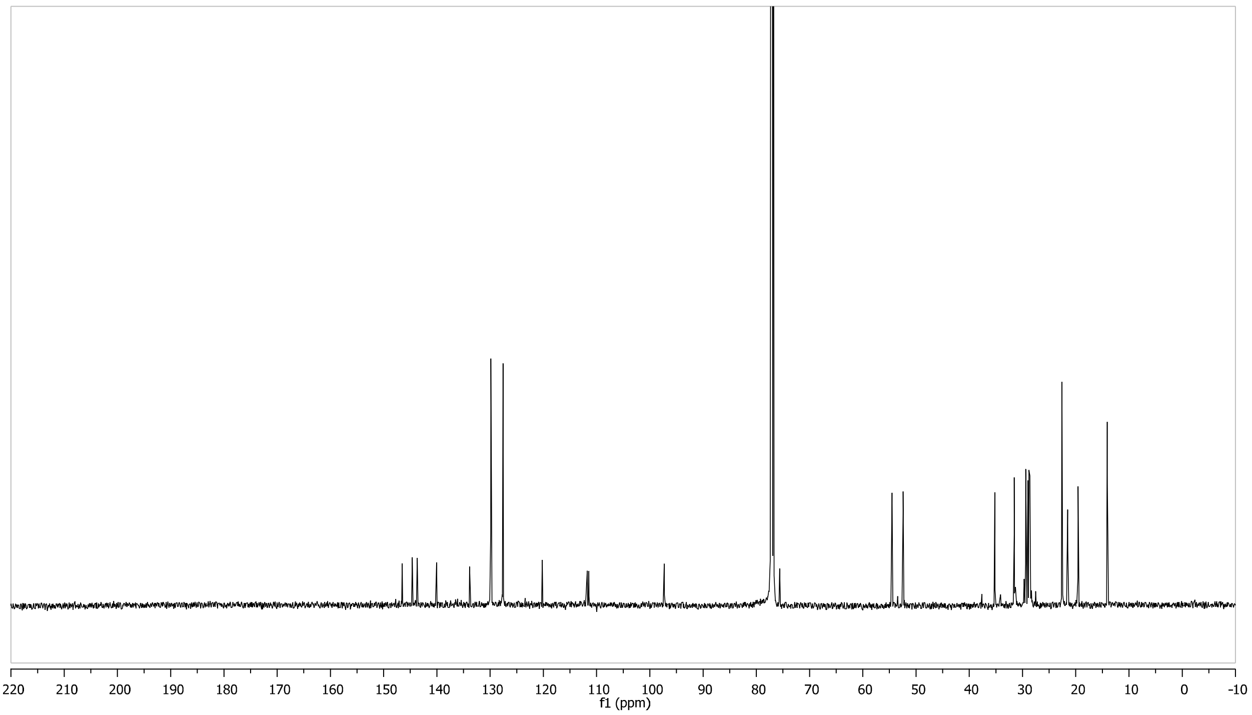
220
210
200
190
180
170
160
150
140
130
120
110
100
90
80
70
60
50
40
30
20
10
0
-10
f1 (ppm)

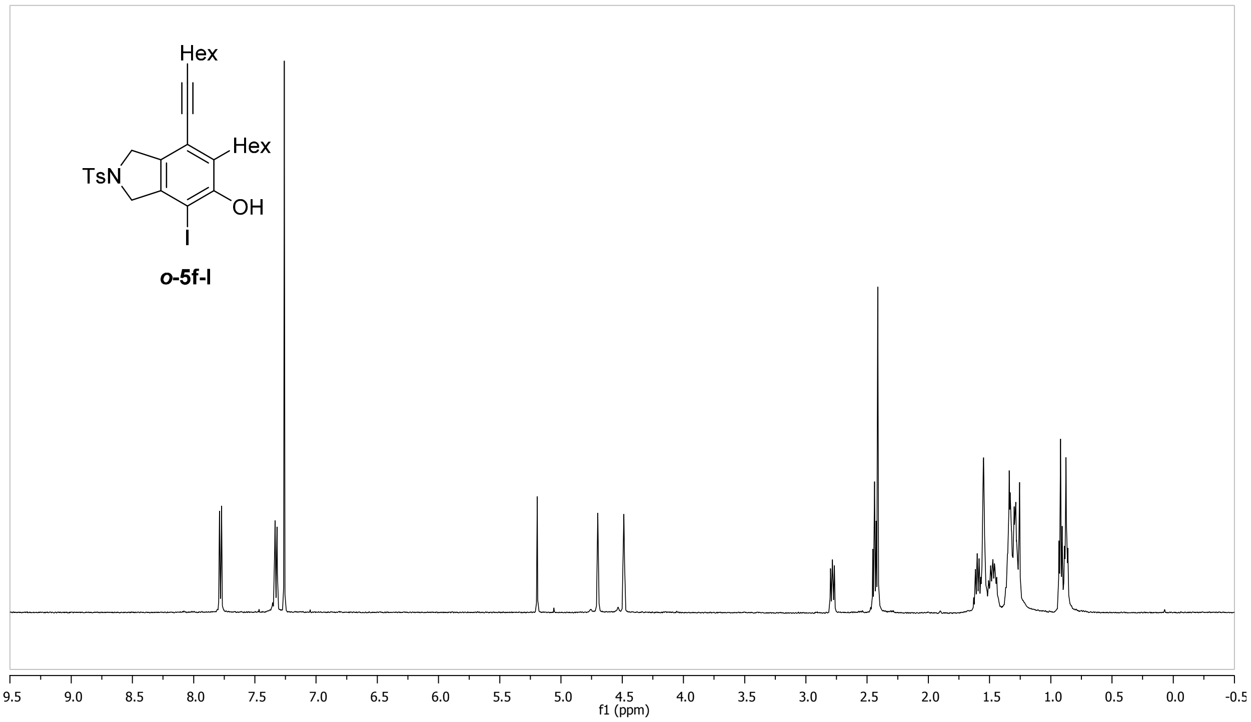
Hex
Hex
TsN
OH
I
o-5f-I
9.5
9.0
8.5
8.0
7.5
7.0
6.5
6.0
5.5
5.0
4.5
4.0
3.5
3.0
2.5
2.0
1.5
1.0
0.5
0.0
-0.5
f1 (ppm)

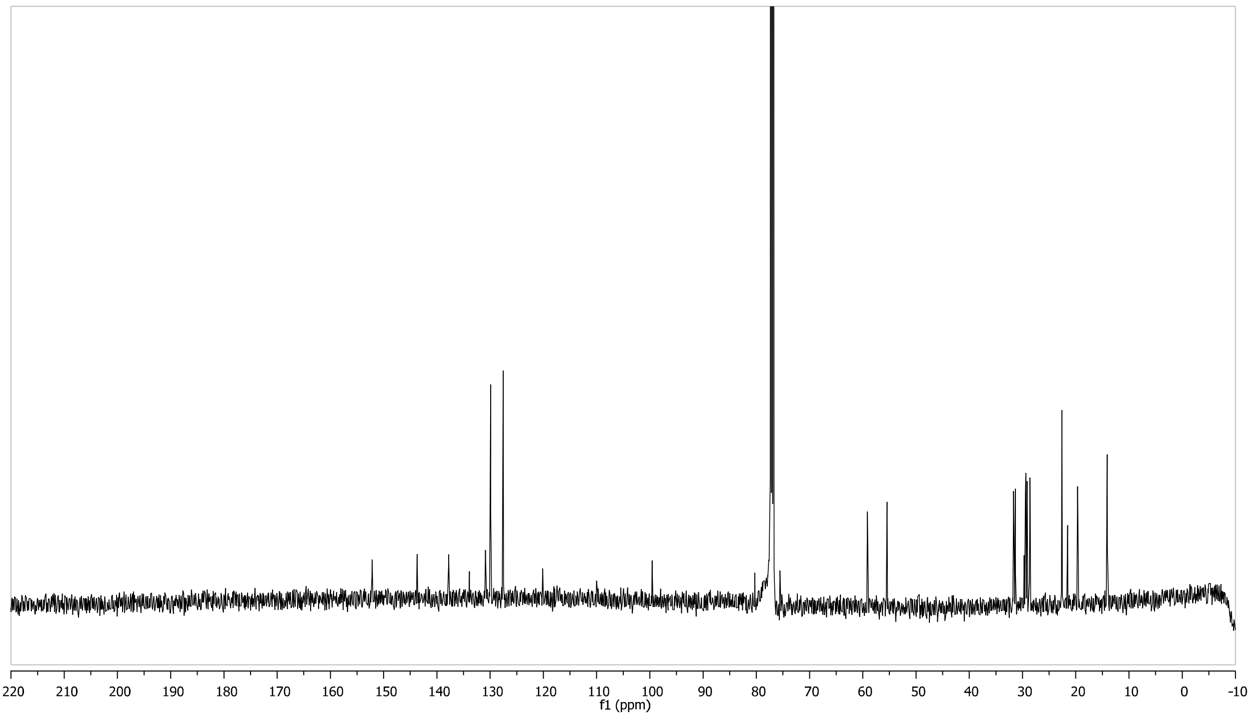

220 210 200 190 180 170 160 150 140 130 120 110 100 90 80 70 60 50 40 30 20 10 0 -10
f1 (ppm)

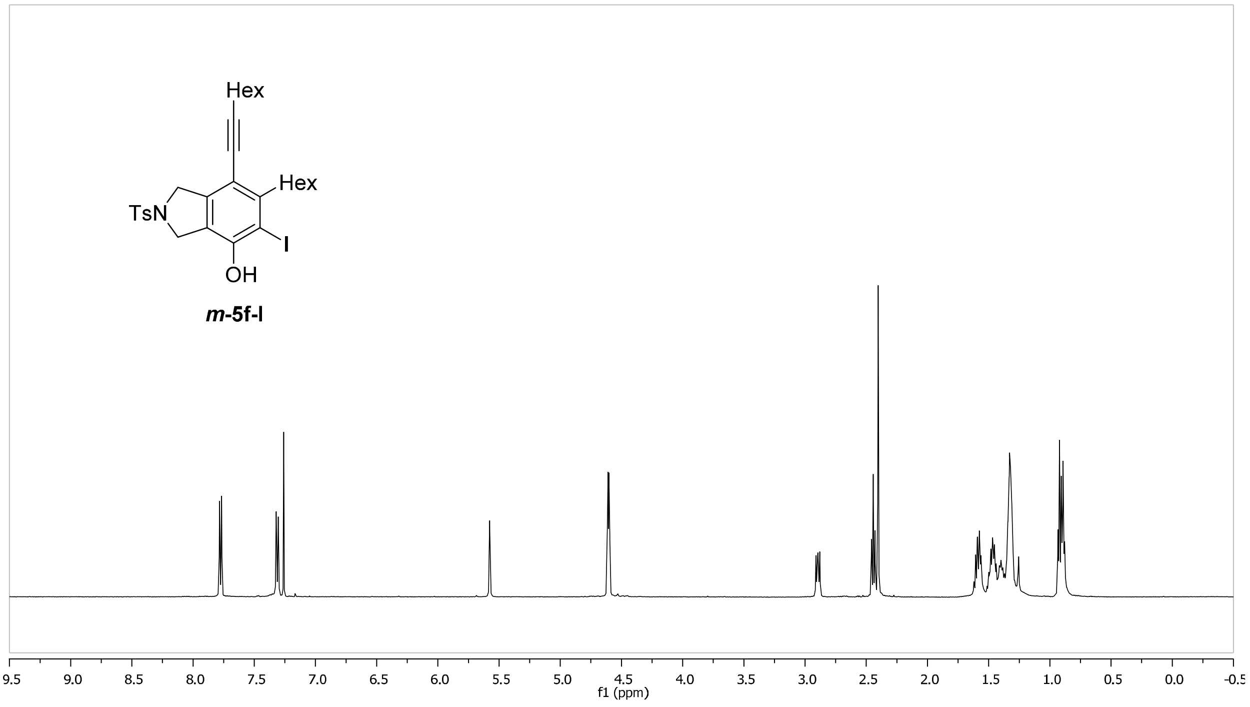

Hex
Hex
TsN
I
OH
m-**5f**-**I**
9.5 9.0 8.5 8.0 7.5 7.0 6.5 6.0 5.5 5.0 4.5 4.0 3.5 3.0 2.5 2.0 1.5 1.0 0.5 0.0 -0.5
f1 (ppm)

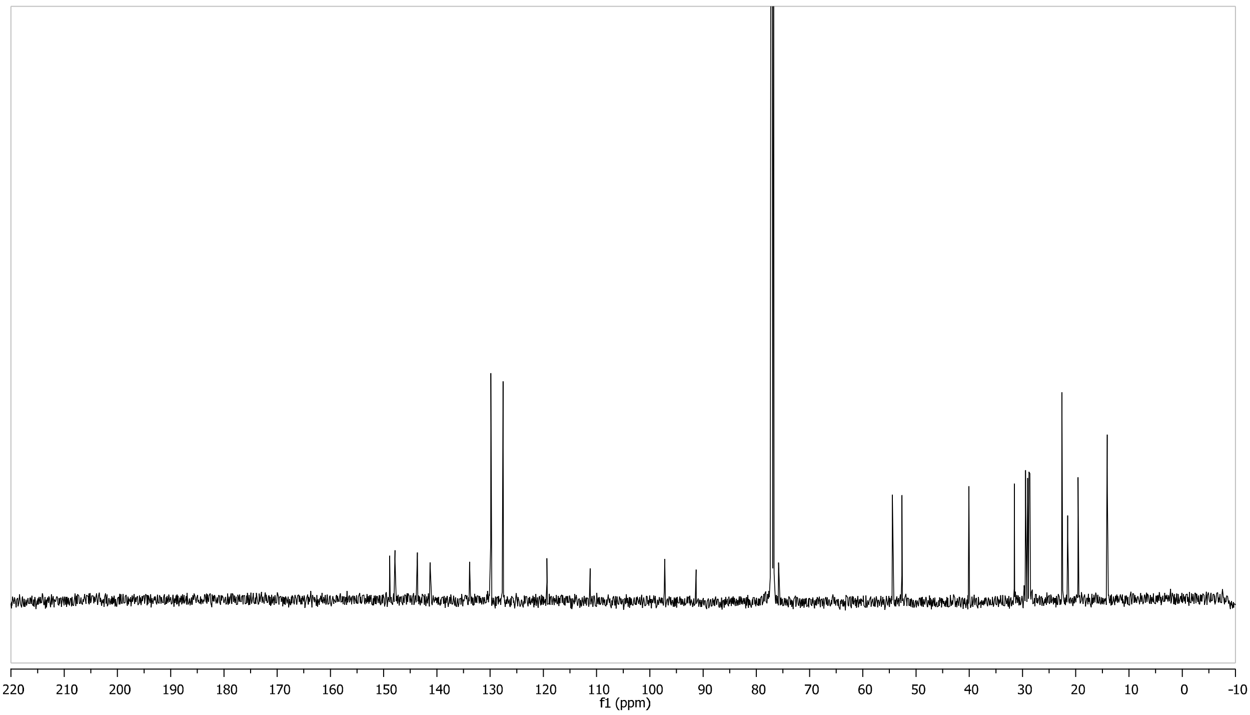
f1 (ppm)

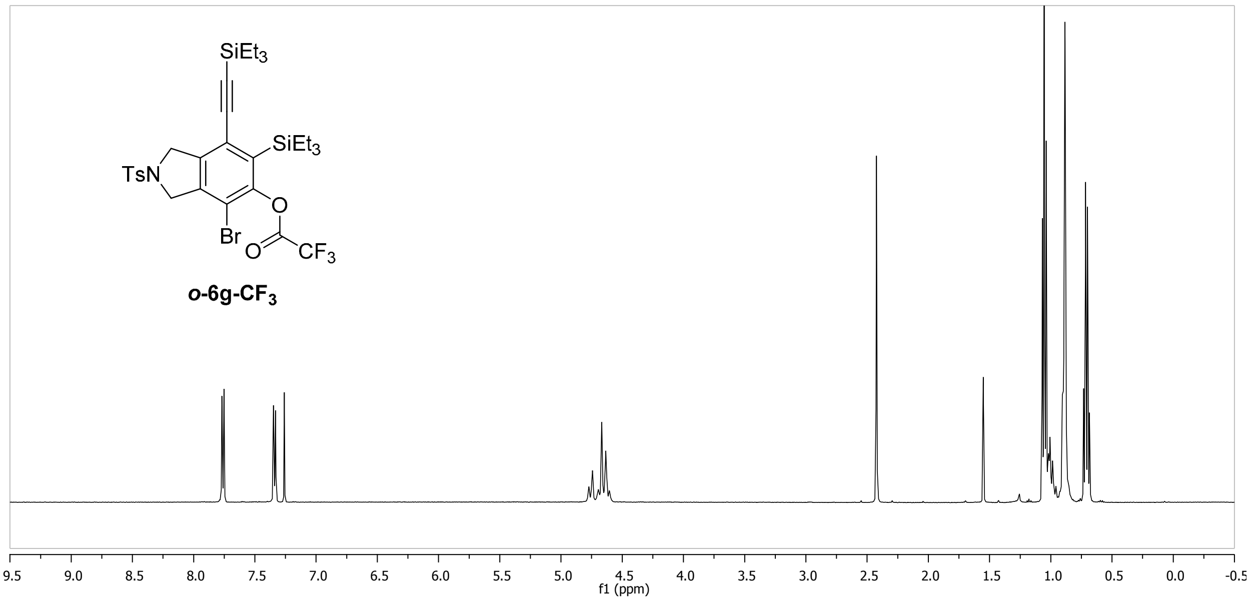
SiEt3
SiEt3
TsN
O
Br
O
CF3
o-6g-CF3
f1 (ppm)

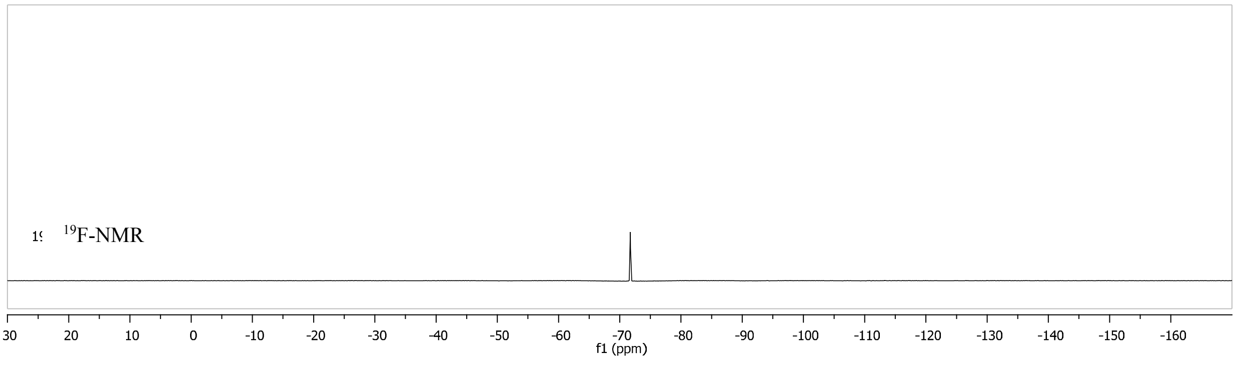
19F-NMR
f1 (ppm)

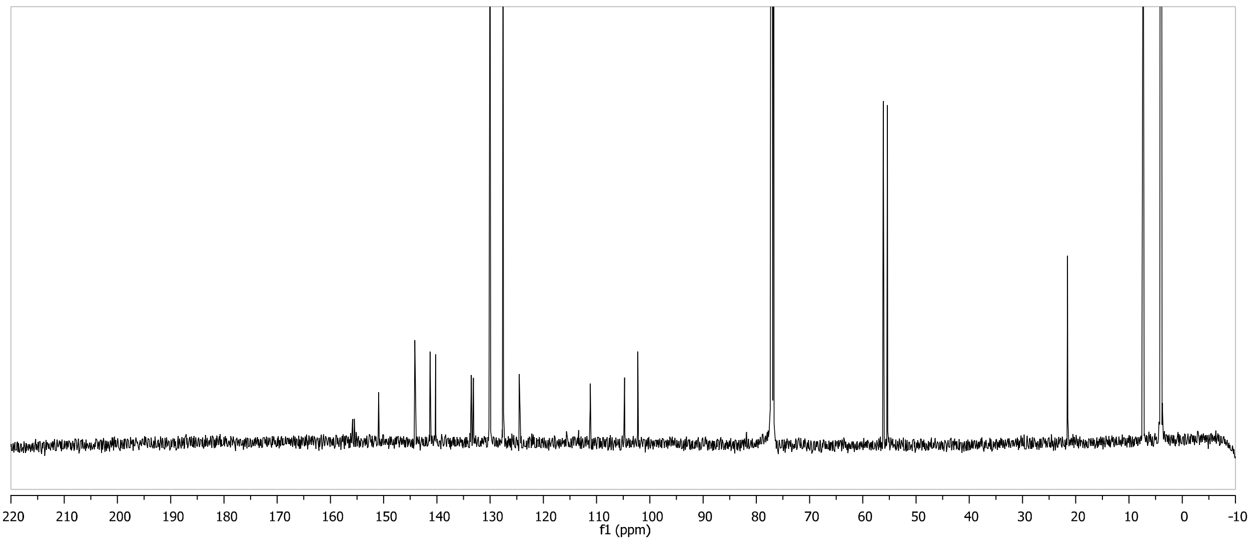

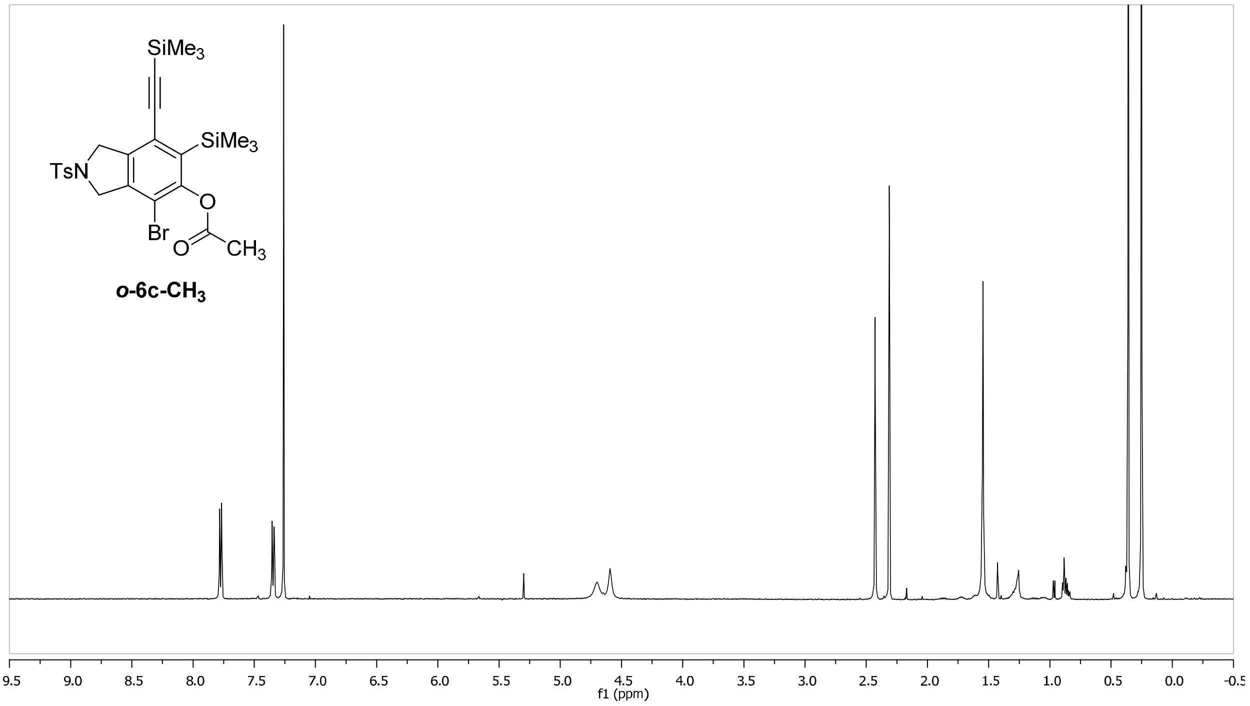

***o*-6c-CH$_3$**

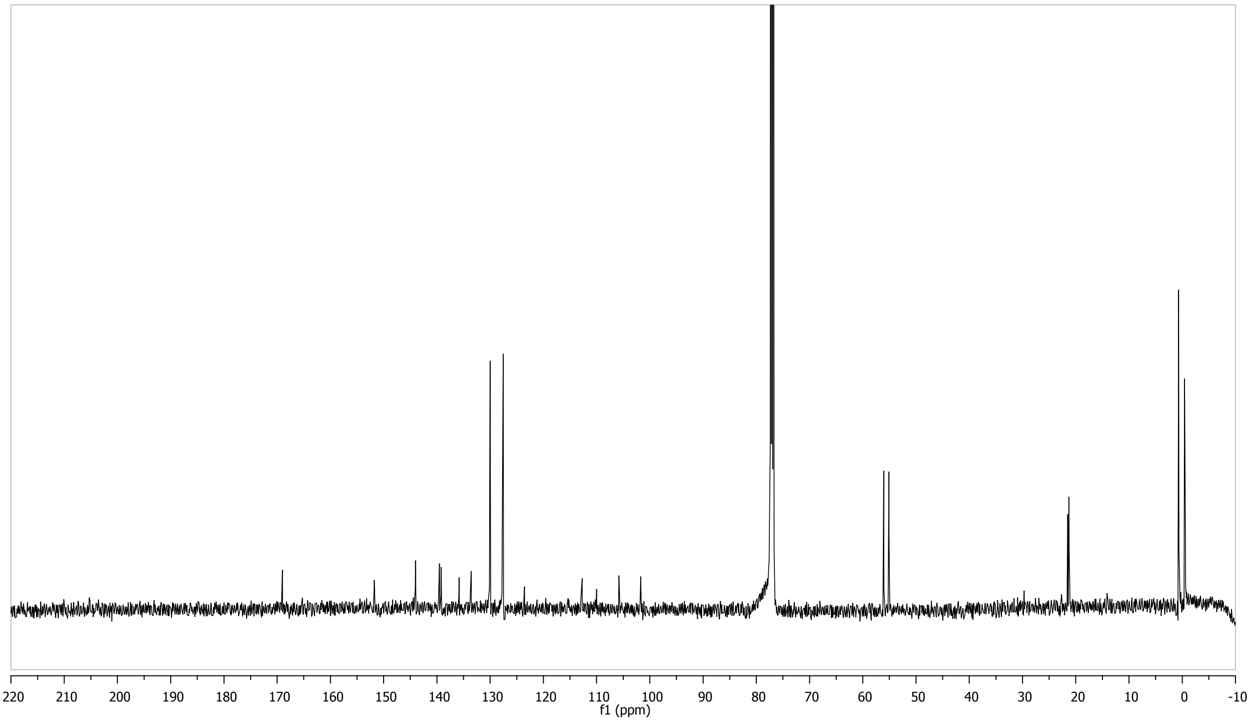
f1 (ppm)
220
210
200
190
180
170
160
150
140
130
120
110
100
90
80
70
60
50
40
30
20
10
0
-10

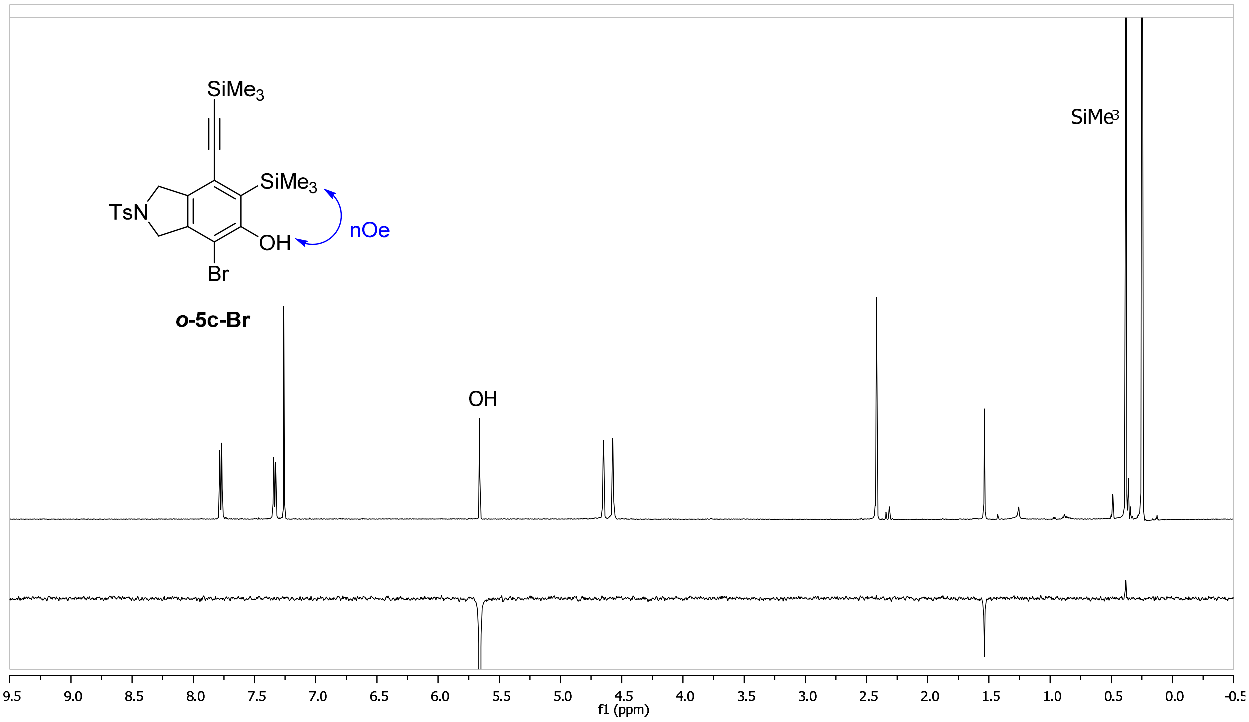
SiMe3
SiMe3
TsN
OH
Br
nOe
o-5c-Br
OH
SiMe3
f1 (ppm)
9.5
9.0
8.5
8.0
7.5
7.0
6.5
6.0
5.5
5.0
4.5
4.0
3.5
3.0
2.5
2.0
1.5
1.0
0.5
0.0
-0.5

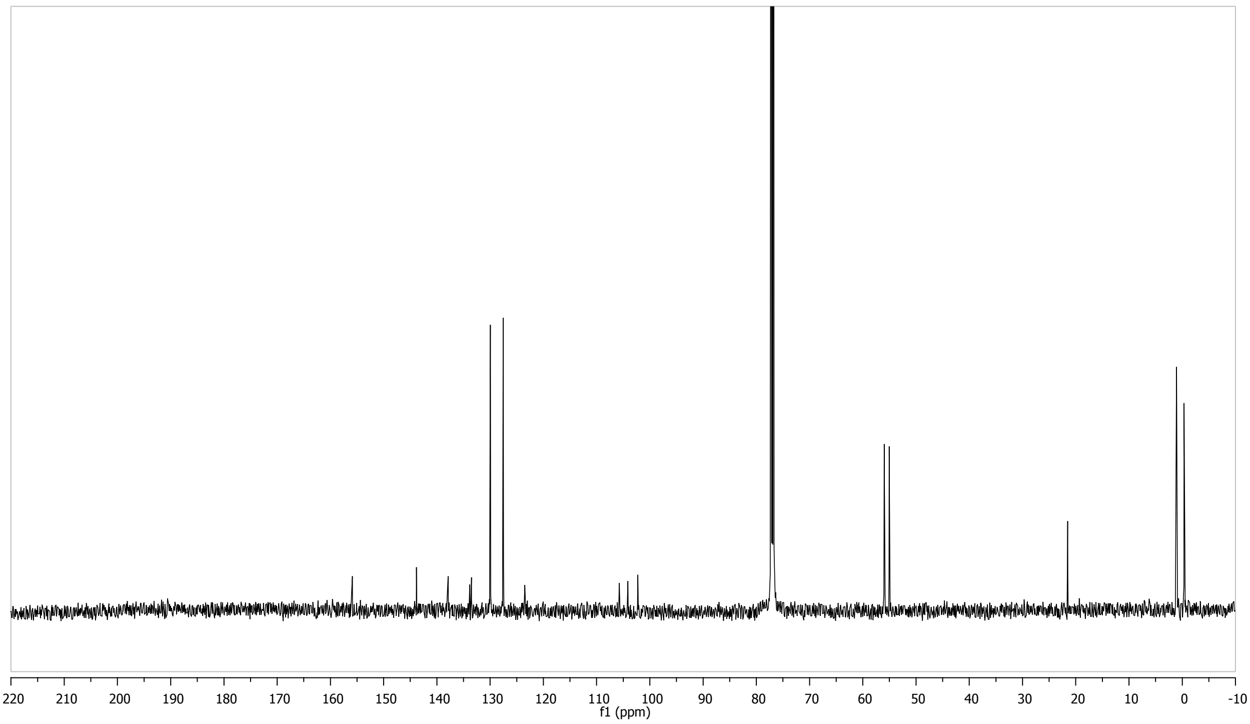
f1 (ppm)

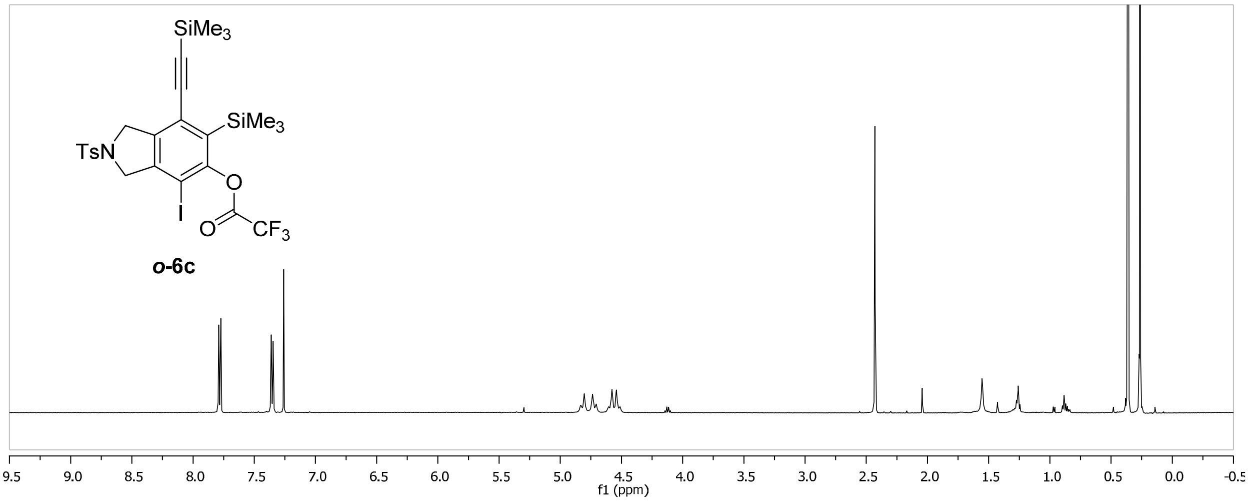
SiMe3
SiMe3
TsN
O
I
O
CF3
o-6c
f1 (ppm)

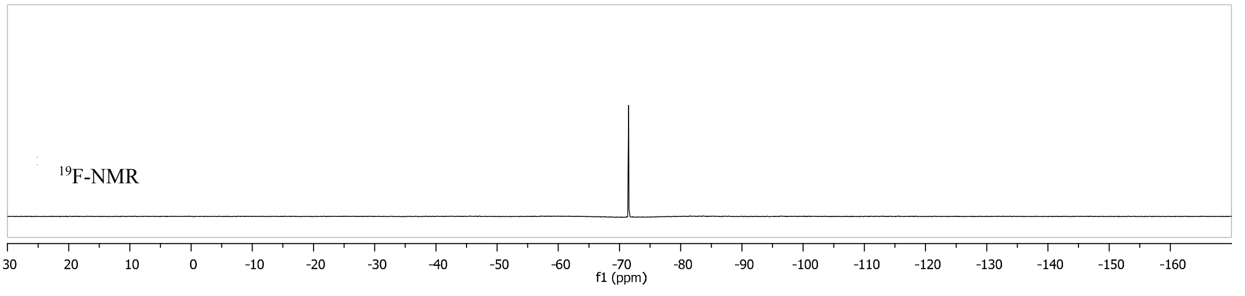
19F-NMR
f1 (ppm)

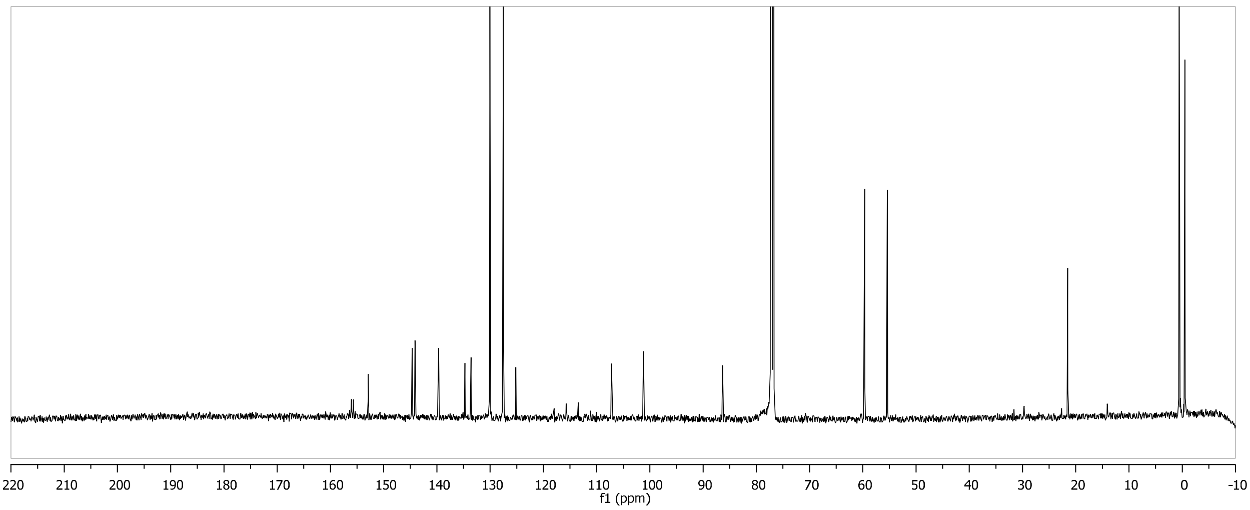
220 210 200 190 180 170 160 150 140 130 120 110 100 90 80 70 60 50 40 30 20 10 0 -10
f1 (ppm)

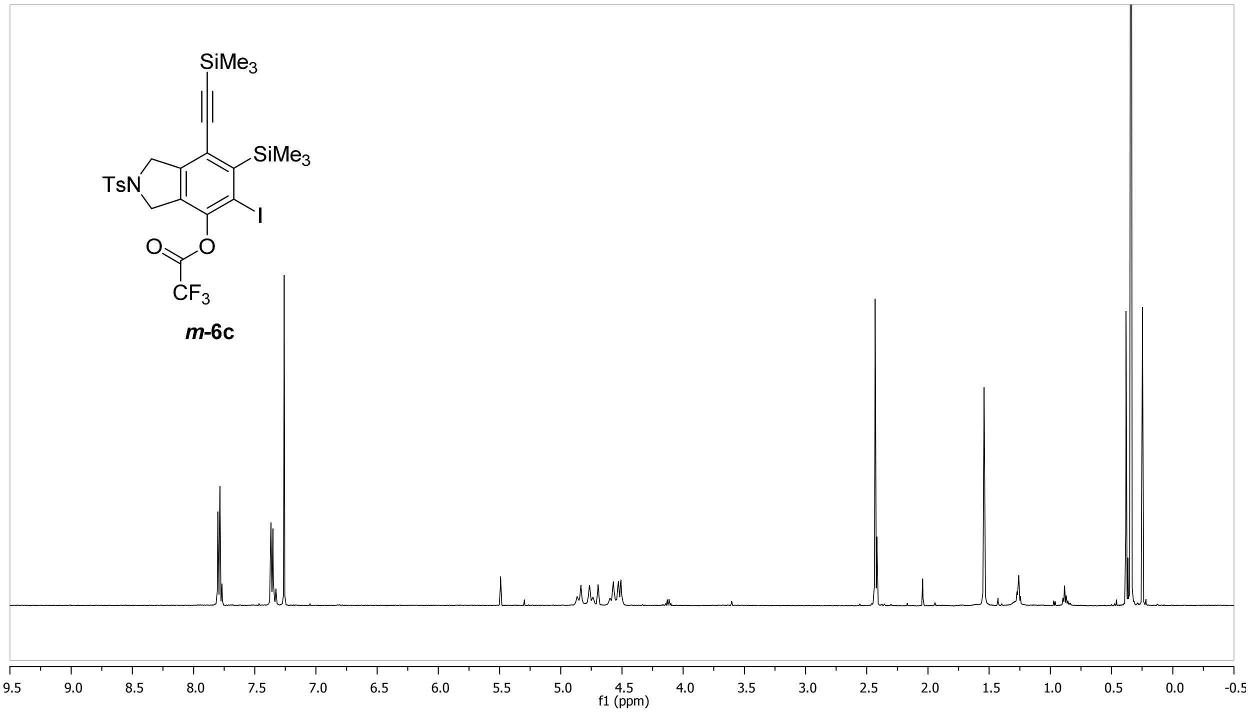
SiMe3
SiMe3
TsN
I
O
O
CF3
m-6c
9.5 9.0 8.5 8.0 7.5 7.0 6.5 6.0 5.5 5.0 4.5 4.0 3.5 3.0 2.5 2.0 1.5 1.0 0.5 0.0 -0.5
f1 (ppm)

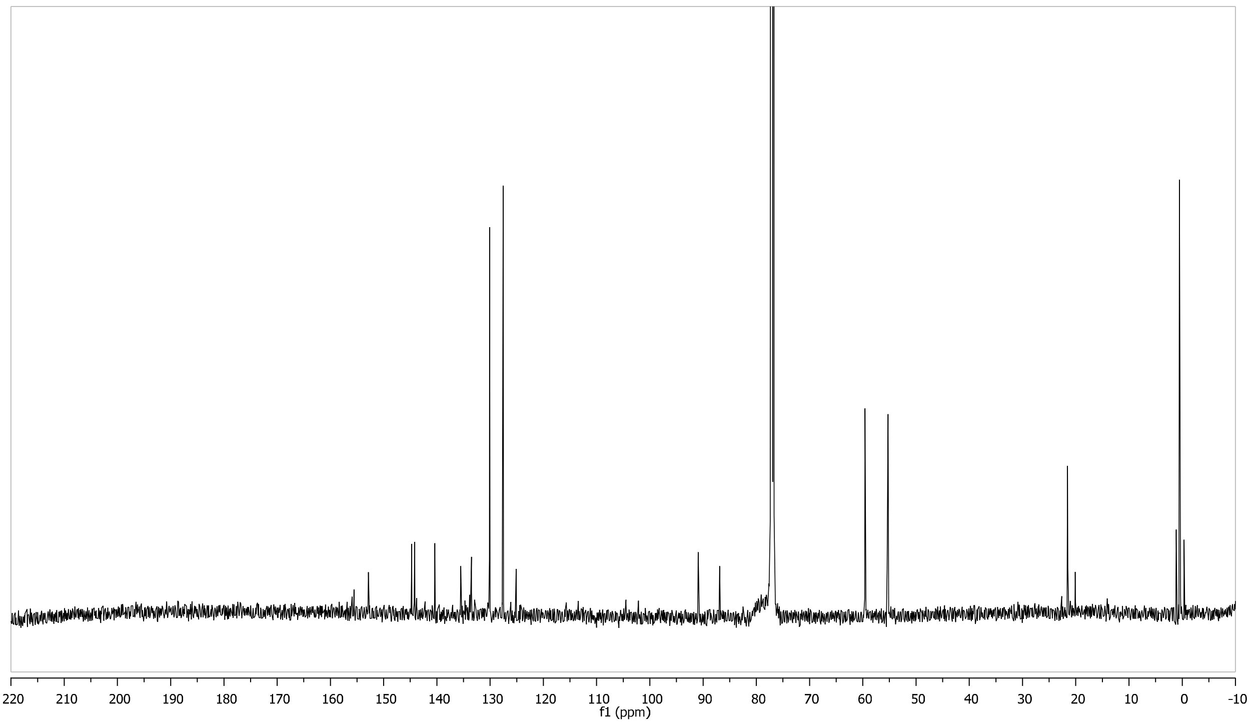
f1 (ppm)

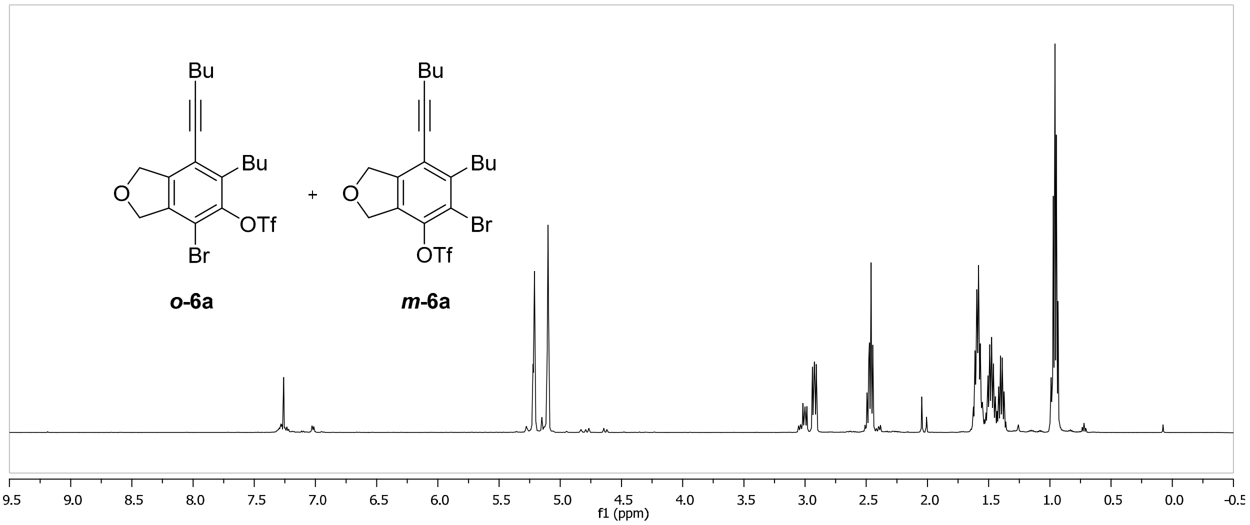
Bu
Bu
O
OTf
Br
+
Bu
Bu
O
Br
OTf
o-6a
m-6a
f1 (ppm)

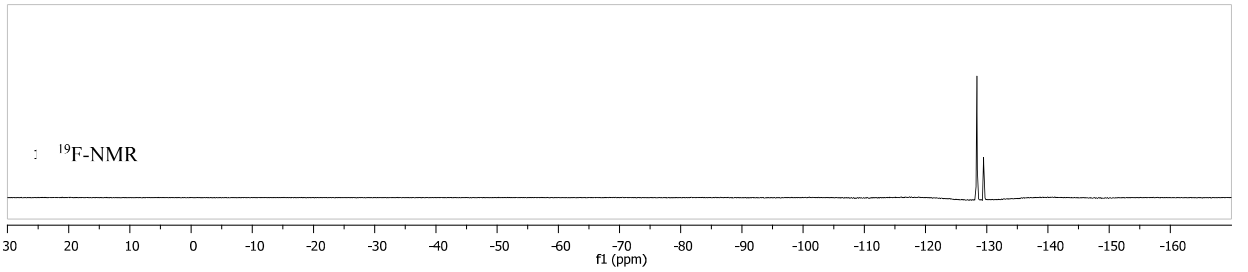
19F-NMR
f1 (ppm)

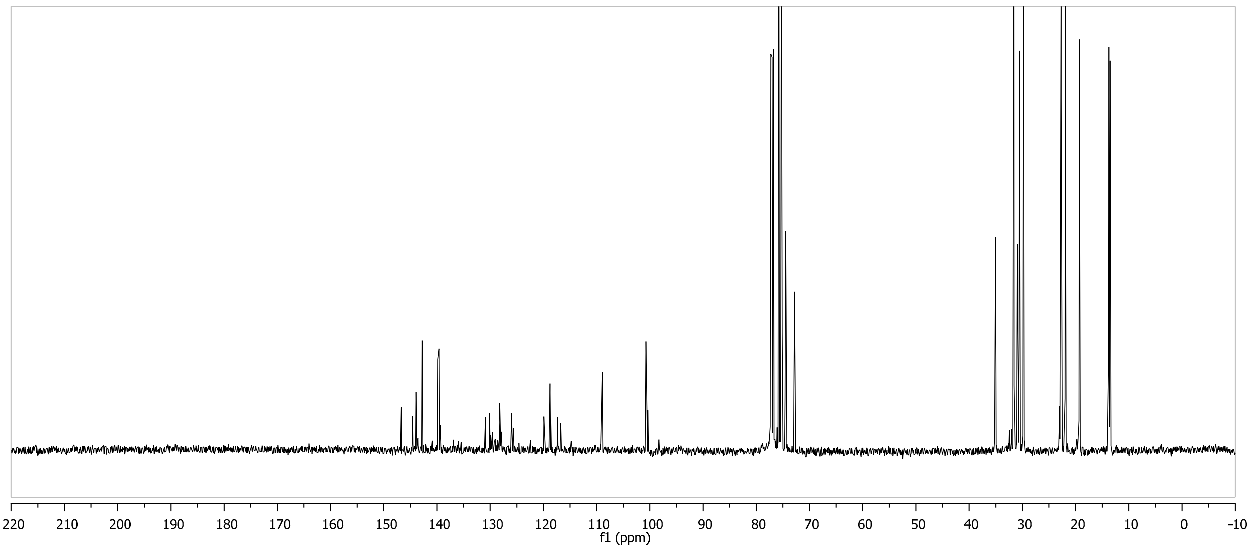
f1 (ppm)

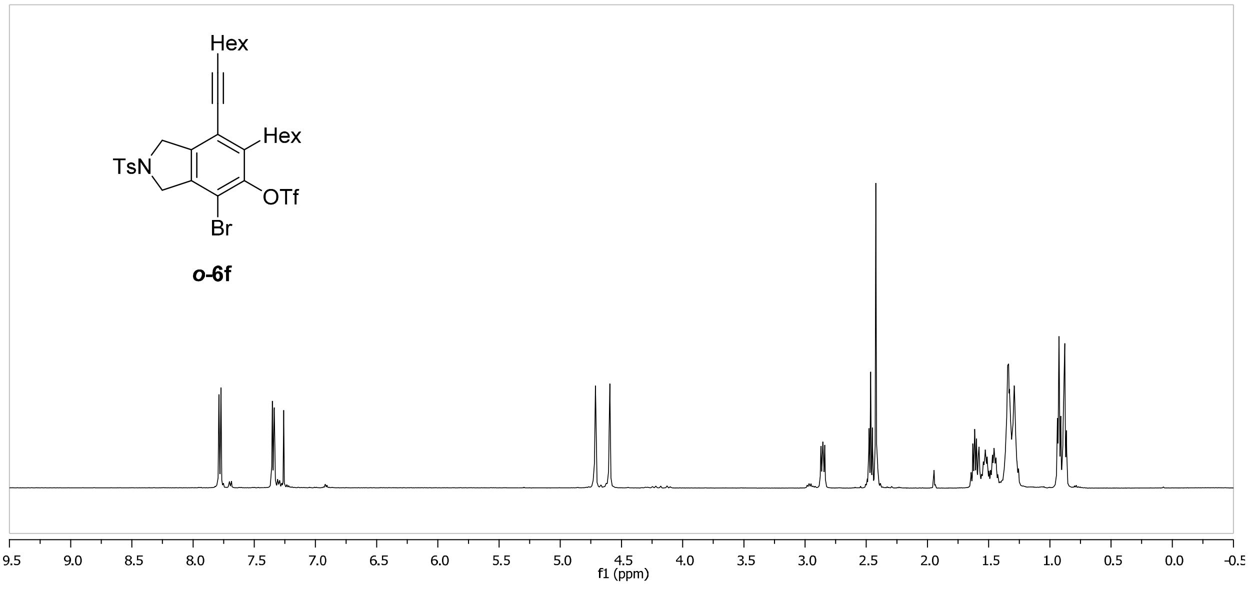
Hex
Hex
TsN
OTf
Br
o-6f
f1 (ppm)

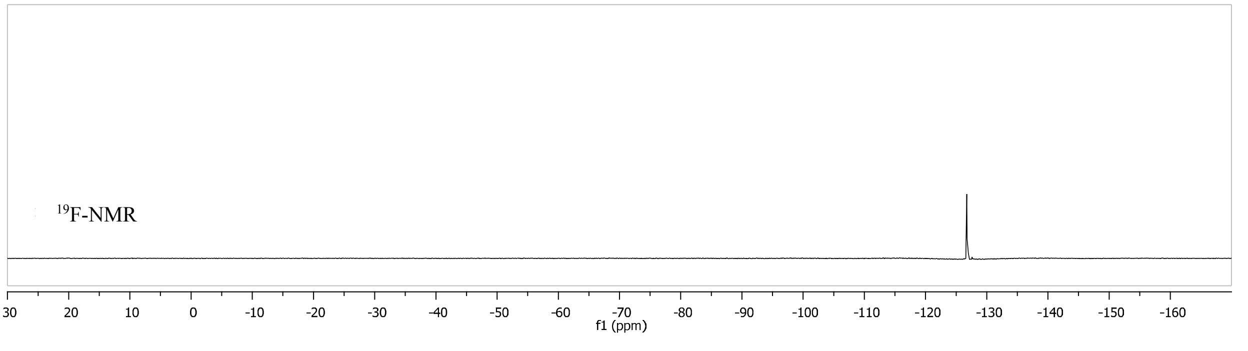
19F-NMR
f1 (ppm)

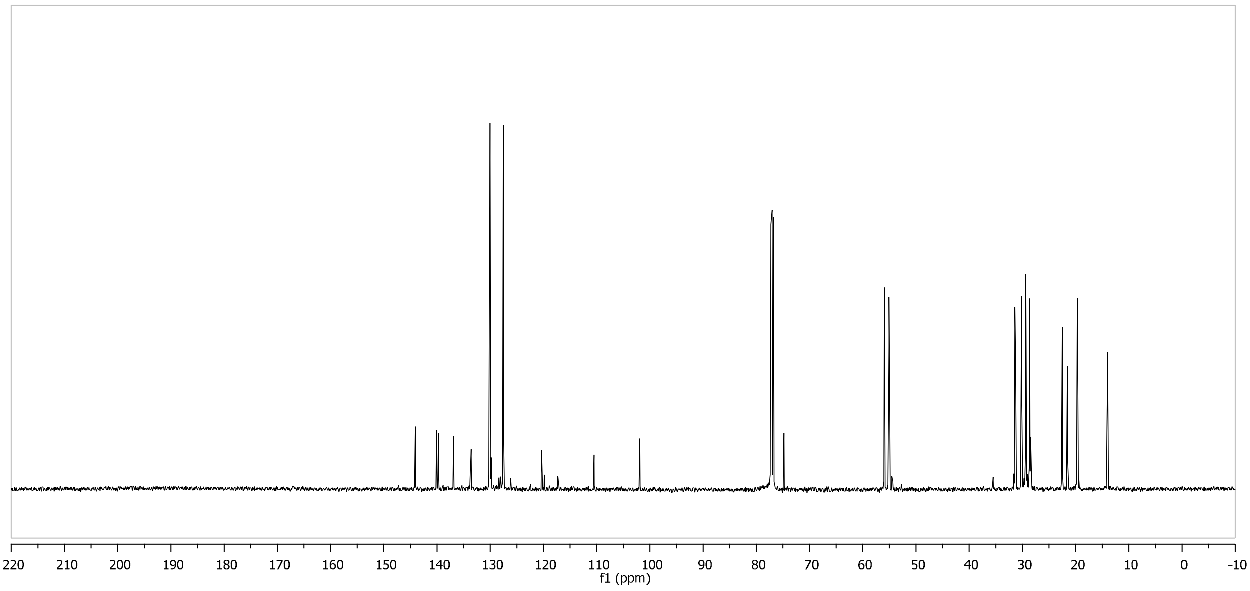
220 210 200 190 180 170 160 150 140 130 120 110 100 90 80 70 60 50 40 30 20 10 0 -10
f1 (ppm)

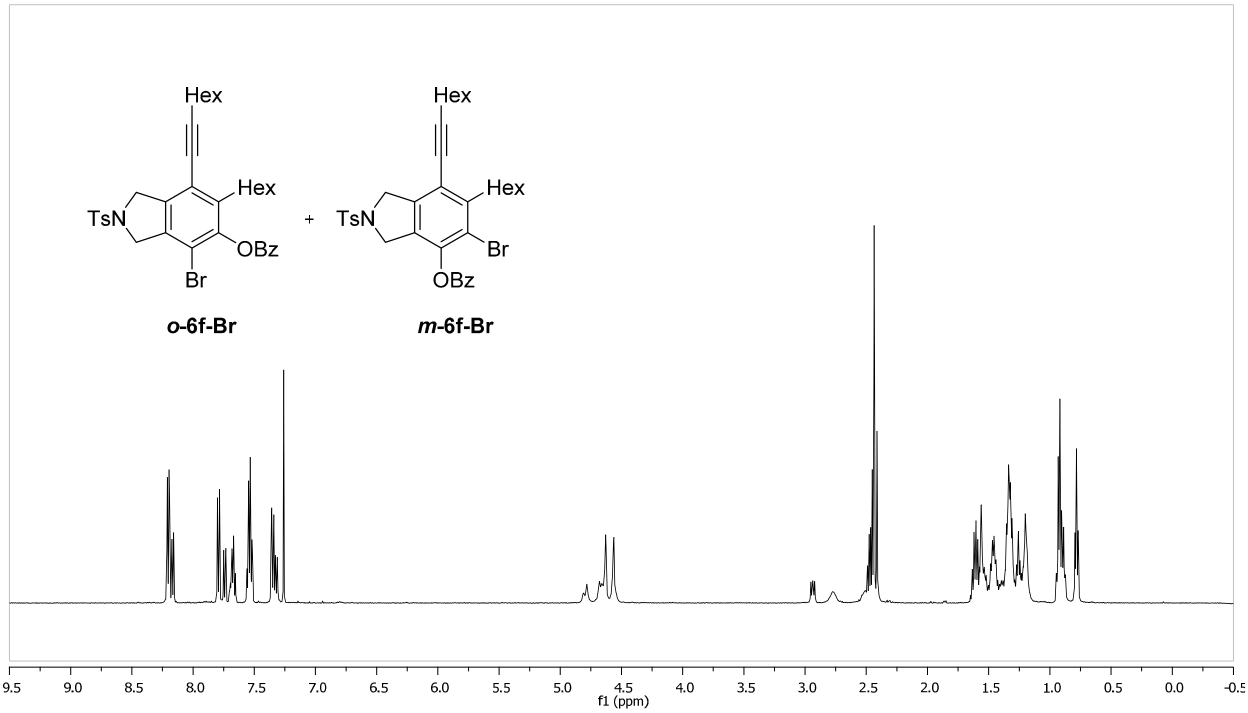
Hex
Hex
TsN
OBz
Br
+
Hex
Hex
TsN
Br
OBz
o-6f-Br
m-6f-Br
9.5 9.0 8.5 8.0 7.5 7.0 6.5 6.0 5.5 5.0 4.5 4.0 3.5 3.0 2.5 2.0 1.5 1.0 0.5 0.0 -0.5
f1 (ppm)

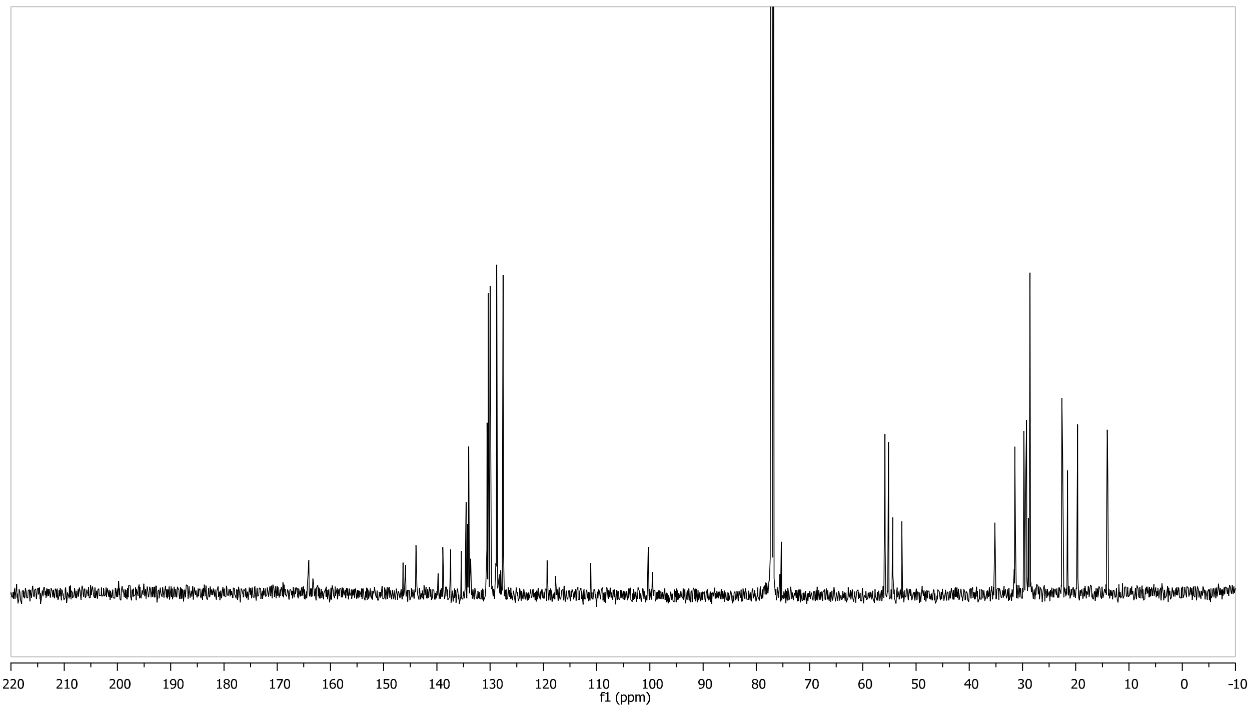
220 210 200 190 180 170 160 150 140 130 120 110 100 90 80 70 60 50 40 30 20 10 0 -10
f1 (ppm)

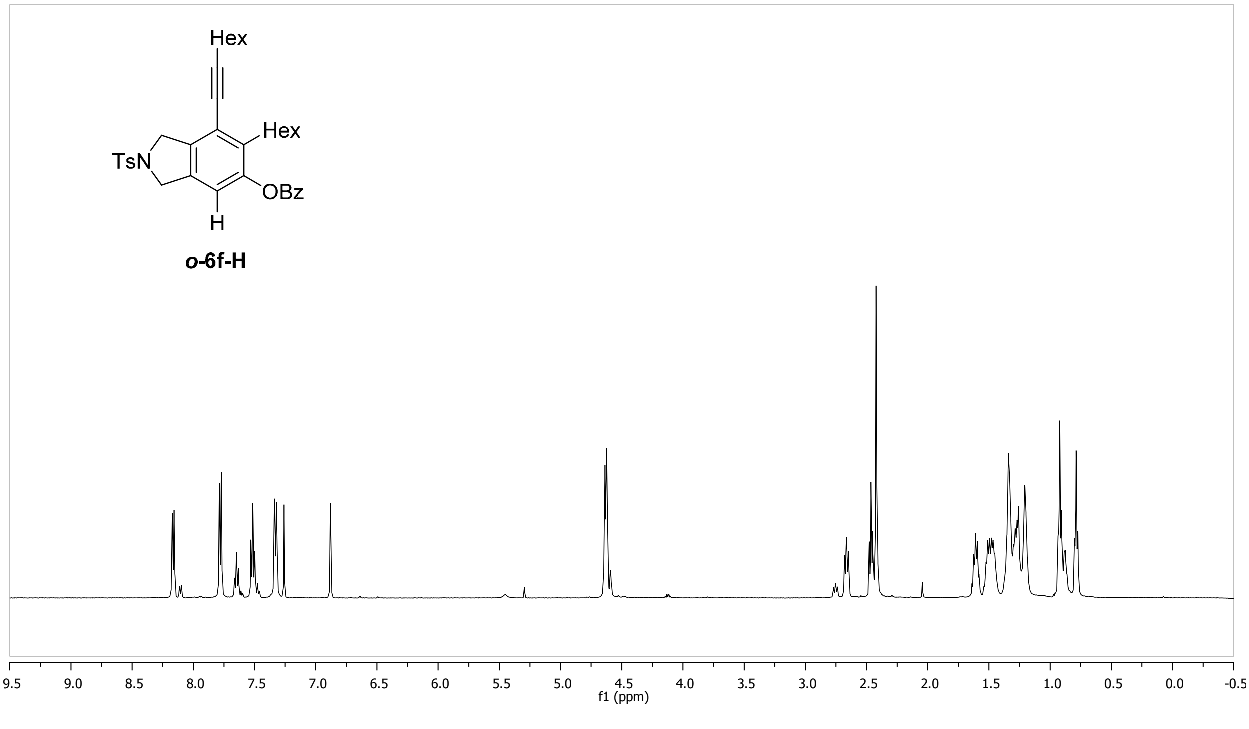
Hex
Hex
TsN
OBz
H
o-6f-H
9.5 9.0 8.5 8.0 7.5 7.0 6.5 6.0 5.5 5.0 4.5 4.0 3.5 3.0 2.5 2.0 1.5 1.0 0.5 0.0 -0.5
f1 (ppm)

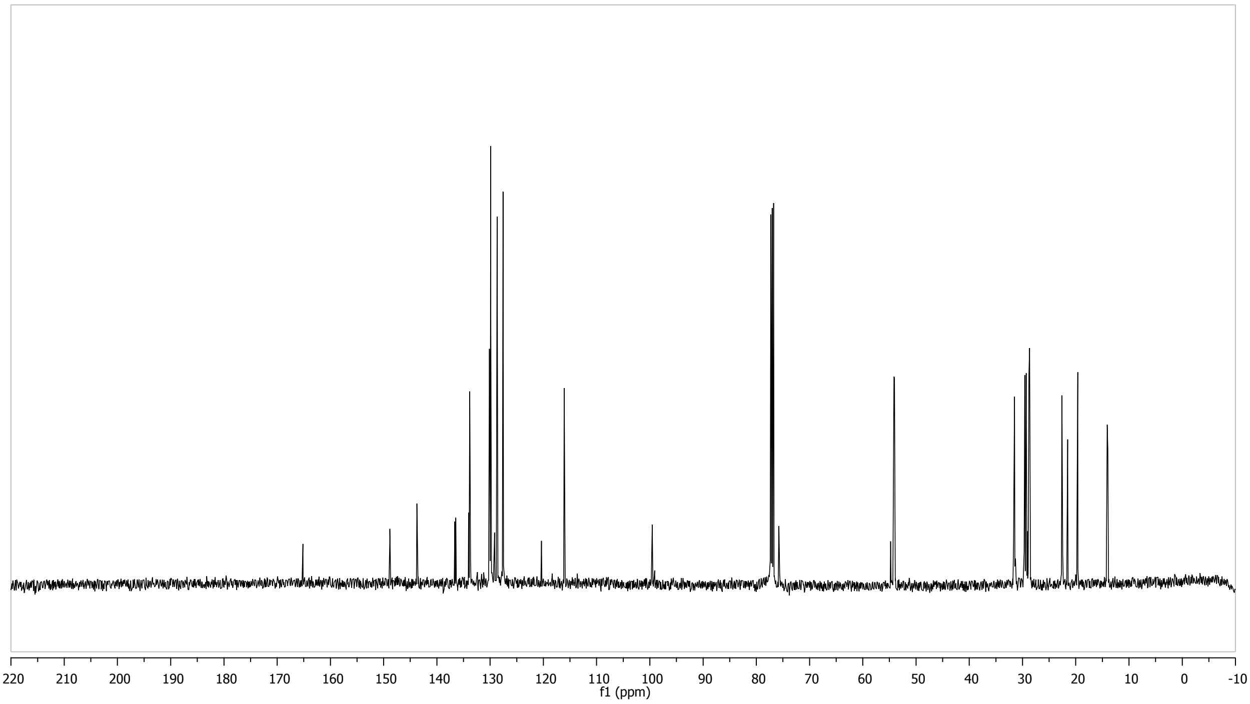
220
210
200
190
180
170
160
150
140
130
120
110
100
90
80
70
60
50
40
30
20
10
0
-10
f1 (ppm)

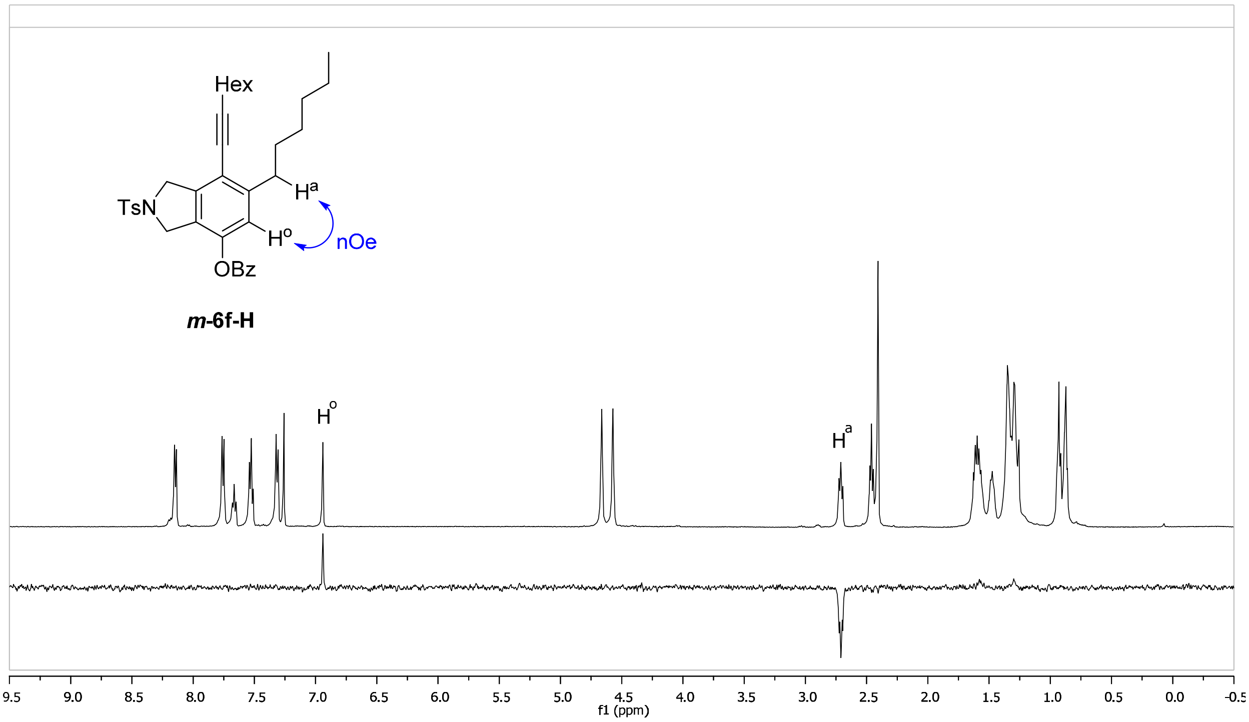
Hex
TsN
Ha
Ho
nOe
OBz
m-6f-H
Ho
Ha
9.5
9.0
8.5
8.0
7.5
7.0
6.5
6.0
5.5
5.0
4.5
4.0
3.5
3.0
2.5
2.0
1.5
1.0
0.5
0.0
f1 (ppm)

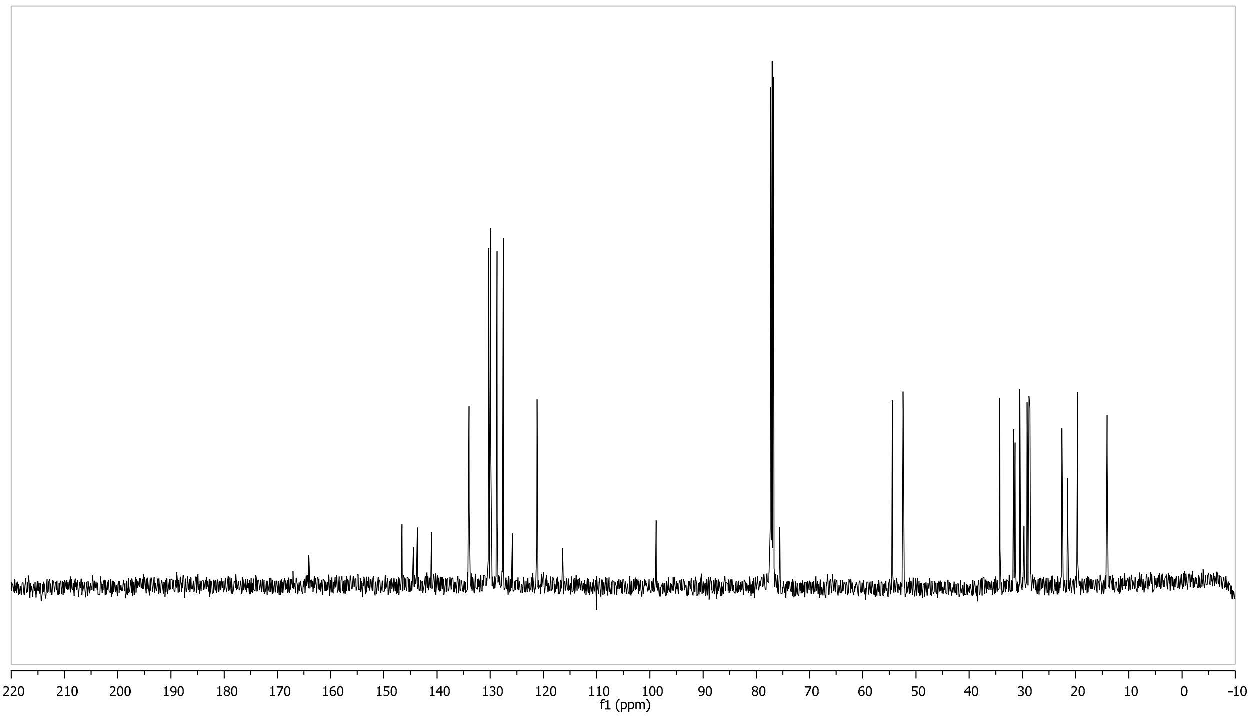

Mulliken Population Analysis

The observed regioselectivity for the reactions of **1d** and **1e** can be further confirmed by the Mulliken Population analysis (but less accurately by NBO analysis) of aryne intermediates **d-INT** and **e-INT**.

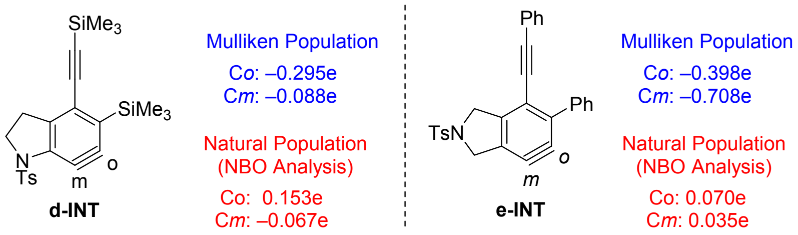

Optimization and population analysis were performed at the B3LYP/6-31+G(d) level of theory.
